# *Curtobacterium* spp. and *Curtobacterium flaccumfaciens*: Phylogeny, Genomics-Based Taxonomy, Pathogenicity, and Diagnostics

**DOI:** 10.3390/cimb44020060

**Published:** 2022-02-11

**Authors:** Peter Evseev, Anna Lukianova, Rashit Tarakanov, Anna Tokmakova, Mikhail Shneider, Alexander Ignatov, Konstantin Miroshnikov

**Affiliations:** 1 Shemyakin-Ovchinnikov Institute of Bioorganic Chemistry, Russian Academy of Sciences, Miklukho-Maklaya Str., 16/10, 117997 Moscow, Russia; a.al.lukianova@gmail.com (A.L.); anna.zem@mail.ru (A.T.); mm_shn@mail.ru (M.S.); 2 Limnological Institute, Siberian Branch of Russian Academy of Sciences, Ulan-Batorskaya Str., 3, 664033 Irkutsk, Russia; 3 Department of Plant Protection, Russian State Agrarian University—Moscow Timiryazev Agricultural Academy, Timiryazevskaya Str., 49, 127434 Moscow, Russia; tarakanov.rashit@mail.ru; 4 Moscow Institute of Physics and Technology, Federal University, Institutskiy per., 9, 141701 Dolgoprudny, Moscow Oblast, Russia; 5 Agrobiotechnology Department, Agrarian and Technological Institute, RUDN University, Miklukho-Maklaya Str., 6, 117198 Moscow, Russia; an.ignatov@gmail.com

**Keywords:** *Curtobacterium*, *Microbacteriaceae*, *Actinomycetales*, prokaryotes, taxonomy, *Curtobacterium* phylogeny, *Curtobacterium* taxonomy, *Curtobacterium* plasmids, *Curtobacterium flaccumfaciens*, *Curtobacterium* pathovars, *Curtobacterium* pathogenicity, phytopathogenicity, virulence factors, *Curtobacterium* diagnostics, PCR diagnostics

## Abstract

The genus of *Curtobacterium*, belonging to the *Microbacteriaceae* family of the *Actinomycetales* order, includes economically significant pathogenic bacteria of soybeans and other agricultural crops. Thorough phylogenetic and full-genome analysis using the latest genomic data has demonstrated a complex and contradictory taxonomic picture within the group of organisms classified as the *Curtobacterium* species. Based on these data, it is possible to delineate about 50 new species and to reclassify a substantial part of the *Curtobacterium* strains. It is suggested that 53 strains, including most of the *Curtobacterium flaccumfaciens* pathovars, can compose a monophyletic group classified as *C. flaccumfaciens*. A genomic analysis using the most recent inventory of bacterial chromosomal and plasmid genomes deposited to GenBank confirmed the possible role of *Microbacteriaceae* plasmids in pathogenicity and demonstrated the existence of a group of related plasmids carrying virulence factors and possessing a gene distantly related to DNA polymerase found in bacteriophages and archaeal and eukaryotic viruses. A PCR diagnostic assay specific to the genus *Curtobacterium* was developed and tested. The presented results assist in the understanding of the evolutionary relations within the genus and can lay the foundation for further taxonomic updates.

## 1. Introduction

Since the definition of the genus *Curtobacterium*, in 1972 [1], the representatives of these microbacteria have been isolated from numerous plants and environmental samples. For a long time, the genus *Curtobacterium* comprised eight validated species, recently accompanied by a number of proposed species and unclassified strain groups. Phytopathogenic strains causing the wilting and rotting of various legumes (*Fabaceae*) and ornamental plants have been established as a separate species, *C. flaccumfaciens*, and further subdivided into several pathovars, depending on the host plants and physiological properties [2,3]. Meanwhile, most described *Curtobacterium* spp. are not known to cause any disease of plants from which they were primarily isolated [4]. Many strains have been isolated as endophytes in sugarcane [5], grapevines [6], maize [7], sorghum [8], tomatoes [9], coffee [10], black peppers [11], strawberries [12], citrus fruits [13], poplars [14] and eucalyptus [15] and have been found in oil brines [5] and marine sediments [7].

Therefore, the role of *Curtobacterium* spp. in plant pathogenesis worldwide and the links between genomic features and virulence with respect to plants used for taxonomic distribution need elaboration and revision.

It is difficult to unravel the structure of the complex genus *Curtobacterium* using data acquired from previous taxonomic studies. Polyphasic studies largely depend on the 16S rRNA gene [16], but despite their usefulness for resolving taxonomic questions in the past, one or a few household genes contain only a limited number of informative characteristics and, thus, can yield phylogenetic trees that lack the resolution to distinguish between closely related species [17]. Classifications based on whole-genome sequences and associated bioinformatic tools provide a significant change in the reliability of phylogenomic trees [17].

New taxonomic technologies often raise the question as to whether discrepancies associated with earlier phylogenetic structures were caused by conflicts between the phenotype, single genes or entire genomes or by data interpretation, such as those between the different algorithms of a taxonomic analysis [18]. In phylogenetic systematics, only monophyletic taxa can be accepted in taxonomic classifications [19].

The present study was designed to provide an improved framework for the classification of the genus *Curtobacterium* based on the principles of phylogenetic systematics applied for genome comparison. A comprehensive sampling of publicly available whole-genome sequences of strains representing the genus *Curtobacterium* was used to construct genome-scale phylogenetic trees and to address the following questions:

(a) Is there any conflict between the phylogenies calculated from whole-genome sequences and the current classification of the genus *Curtobacterium*?
(b) Which species within the genus *Curtobacterium* need to be revised because they are evidently nonmonophyletic?

Another important question concerns the relatedness between the mechanisms of pathogenicity of curtobacteria and their taxonomy and phylogeny. The genomes of *Curtobacterium* plasmids have been shown to contain possible virulence genes, and the effect of mobile elements and gene exchange could reveal the ways of the emergence of pathogenic strains and pathovars [20,21,22,23]. In this study, *Curtobacterium* plasmids were studied in the context of the genomics of pathogenicity.

In addition, this research concerns the development of existing diagnostic methods to better correspond to the *Curtobacterium* taxonomy and genomic data. The abbreviations in the text below are as follows: *C.*—*Curtobacterium*, *C. f.*—*Curtobacterium flaccumfaciens*, *C. fpf*—*flaccumfaciens* pv. *flaccumfaciens* and *C. f.* pv.—*Curtobacterium flaccumfaciens* pathovars.

## 2. Materials and Methods

### 2.1. Genomes’ Annotation and Comparison

Bacterial chromosomal and plasmid genomes were downloaded from the NCBI Genome database [24]. Chromosomal genomes were annotated with Prokka [25], using the default settings of Prodigal [26] for finding open reading frames (ORFs) and Barrnap [27] for rRNA gene detection. The plasmid genomes were annotated manually, using Geneiuos [28], Glimmer [29] and Prodigal [26] for ORF detection. Functional assignments of plasmid genes were made using a BLAST homology search [30] on the NCBI nr/nt database and custom databases using a HMM-HMM search with HHpred [31] and Phyre2 [32], and using an InterPro [33] search.

A genome sequence comparison and a visualisation of the annotated plasmid genomes were made with EasyFig [34], applying TBLASTX [30]. 

### 2.2. ANI Calculation and Clustering

The average nucleotide identity (ANI) was calculated using OrthoANIu [35]. The data obtained were clustered using the Phylogeny.fr server [36], applying the BIONJ algorithm [37] to the distance matrix. The BIONJ dendrogram was visualised using the iTol server [38].

### 2.3. Phylogenetic Analysis of rRNAs, Ribosomal Proteins, gyrB, parE, rpoA and rpoB

Genes sequences of the rRNAs, ribosomal proteins, *gyrB*, *parE*, *rpoA* and *rpoB* were extracted from the annotated genomes and aligned with MAFFT [39] with L-INS-i algorithms and other default settings. The concatenation of the sequences was carried out using Geneious tools [28]. Phylogenetic trees were constructed using maximum likelihood methods implemented in MEGA X [40] and RAxML [41] software packages, applying Tamura-Nei [42] and General Time Reversible (GTR) [43] nucleotide substitution models.

### 2.4. Phylogenetic Analysis of Core Genome

The core genes’ concatenated alignment was obtained with the Panaroo pipeline [44] using Prokka annotated genomes, (--core_threshold = 0.95 --aligner clustal) settings, and with other parameters set to default. Phylogenetic inference was performed by RAxML using the GTR-CAT nucleotide substitution model [41].

### 2.5. Protein Structure Modelling

Protein structure modelling was performed in two steps. First, the structure was predicted using AlphaFold 2.0 [45] with default settings, I-TASSER [46,47] with default settings, Phyre2 [32] in intensive modelling mode and Rosetta [48] using RoseTTAFold modelling mode [49]. The accuracy of prediction of the best-scoring models obtained by the methods listed above was estimated using ModFold8 [50], and the model with the highest global model quality score was used for the refinement of the structure using ReFOLD3 [51] in the next step. The quality of the prediction of the resulting structure was also estimated by ModFold8 [50]. Similar experimentally obtained structures were found by the HHpred [31], Phyre2 [32] and I-TASSER [47] servers. The superimposition and visualisation of the structures were achieved using PyMOL v.2 [52].

### 2.6. Primer Design

The phylogenetic analysis showed that the 23S rRNA sequence could be used to differentiate *Curtobacterium* from other genera with fairly good reliability. To develop primers, the 23S rRNA sequences of all the available *Curtobacterium* strains and a set of unrelated strains were aligned using MAFFT [39]. Then, following the alignment, a conserved and unique plot for *Curtobacterium* was manually determined. Oligonucleotides for the amplification of this region were optimised using Primer3Plus [53].

### 2.7. Bacterial Strains and Growth Conditions

A total of 81 bacterial strains, listed in Table S1, were used in the research. Twenty-two strains were purchased from the All-Russian Collection of Microorganisms (Pushchino, Moscow Oblast, Russia). Another 23 strains were isolated from the affected plants using a semi-selective medium for the isolation of *Curtobacterium* [54]. The remaining strains were taken from the collection of phytopathogenic bacteria of the Laboratory of Molecular Bioengineering of the Institute of Bioorganic Chemistry of the Russian Academy of Sciences (Moscow, Russia), used in previous research [55,56].

Routine cultivation of the strains was carried out on YD medium (yeast extract—10 g/L, dextrose—20 g/L, and agar 15 g/L) at a temperature of 28 °C. Long-term storage of the strains was carried out at −80 °C in 30% glycerol.

### 2.8. DNA Isolation

A GeneJET Genomic DNA Purification Kit (Thermo Scientific, Waltham, MA, USA) was used to isolate genomic DNA following the manufacturer’s instructions. The concentration of purified DNA was measured using a NanoProteometer N60 spectrophotometer (NanoProteometer, Munich, Germany). On average, the DNA amount in the samples was ~20 ng.

### 2.9. Polymerase Chain Reaction (PCR)

Conventional PCR was performed using a BioRad T100 ThermalCycler (Bio-Rad, Hercules, CA, USA) in a volume of 25 μL. Each reaction contained five μL of ScreenMix (Evrogen, Moscow, Russia), 0.3 mM of each primer, and 20 ng of template DNA.

The thermal cycling mode was 94 °C for 300 s, followed by 30 cycles of 94 °C for 30 s, 65 °C for 10 s, and 72 °C for 20 s, with a circuit extension at 72 °C for 5 min. PCR products were visualised by electrophoresis (1.5% agarose gel with the addition of ethidium bromide and 1× TAE buffer). A 1-kb ladder length marker (Evrogen) was used to determine the size of the DNA fragments.

### 2.10. Test Plasmid Construction

To construct a test plasmid, the target sequence was first amplified in the Cff VKM Ac-1923 (← DSM 20129) strain. After the resulting PCR, the product was purified using the Qiagen MinElute PCR Purification kit (Qiagen, Dusseldorf, Germany) and ligated into the pDrive vector (Qiagen) using the Qiagen PCR Cloning kit. The correctness of the insertion was checked using Sanger sequencing.

### 2.11. qPCR Conditions

PCR was performed in a final volume of 10 μL. The reaction mixture included 2 μL of Evrogen qPCRmix-HS SYBR, 0.3 mM of each primer, and 20 ng of template DNA. The final concentration of dNTP was 0.12 mM, and the concentration of magnesium was 3 mM. Thermal cycling was performed on a LightCycler 96 (Roche, Basel, Switzerland) in the following mode: 94 °C for 300 s, then 45 cycles of 94 °C for 10 s, 65 °C for 10 s, and 72 °C for 10 s. All experiments were carried out in duplicate per run and repeated twice. Thus, there were four technical replicates. The processing of the amplification curves and calculation of the threshold cycles were carried out using Roche software. Reactions with water were used as a negative control and with a test plasmid as a positive control.

For the experiment to determine the detection sensitivity, tenfold dilutions of the test plasmid and genomic DNA Ac-1923 (← DSM 20129) were prepared. PCR was performed with each dilution in the same way as described above.

## 3. Results

### 3.1. Curtobacterium Genomes in GenBank Database, Mislabelled Strains, and Curtobacterium flaccumfaciens Pathogenic Strains

**Figure 1:**
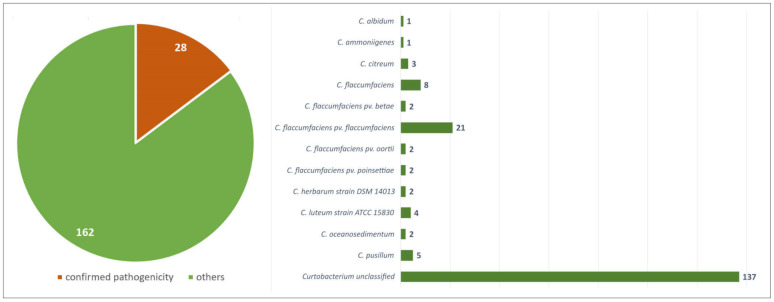
Statistics on 190 *Curtobacterium* genomes deposited in the NCBI database as of October 2021.

As of October 2021, the NCBI Genome database [24] contained drafts and complete genomes of 191 strains attributed as *Curtobacterium*, including two metagenome assemblies. Most of the strains were unclassified on the species level; 35 strains were classified as *Curtobacterium flaccumfaciens*, including 27 pathovars; and seven strains were classified as representatives of seven other *Curtobacterium* species (*C. albidum*, *C. ammoniigenes*, *C. citreum*, *C. herbarum*, *C. luteum*, *C. oceanosedimentum*, and *C. pusillum*). The preliminary analysis demonstrated that one of these strains, *Curtobacterium* S6, had the lowest average nucleotide identity (ANI) value, i.e., about 69% of all the other *Curtobacterium* strains. The phylogenetic analysis using 16S rRNA and 23S rRNA sequences of the closest homologues belonging to different *Microbacteriaceae* genera placed strain S6-1 distantly from the *Curtobacterium* clade (data not shown). Seemingly, this strain has been misclassified and might possibly represent a novel genus. Thus, only 190 genomes can be considered as *Curtobacterium* spp. (Figure 1).

It is difficult to estimate the number of *C. flaccumfaciens* pathogenic strains deposited in the genome database confidently, since the majority of the deposited strains have not been tested in biological experiments. However, the data available in the literature makes it possible to distinguish at least 28 pathogenic strains (Table 1).

### 3.2. ANI Analysis

Although the current taxonomy still relies on the classification system designed by Carolus Linnaeus, the use of criteria such as the genome index of the average nucleotide identity (ANI) can assist taxonomy [57,58,59]. The proposed minimal standards [60] and the regular practice of species delineation include ANI calculations and a multilocus phylogenetic analysis (MLPA). These standards apply to the ANI cut-off score of >95%, which indicates that strains belong to the same species [61,62,63]. The ANI data correlate well with DNA–DNA hybridisation results, and the recommended cut-off point of 70% DDH for species demarcation corresponds to 95% ANI [64,65]. A number of software packages can be used for calculations of ANI, including orthoANIu [35], Jspecies [65], ANI calculator [66], FastANI [62] and Gegenees [67]. Calculations with different packages can yield slightly different results [61].

**Figure 2:**
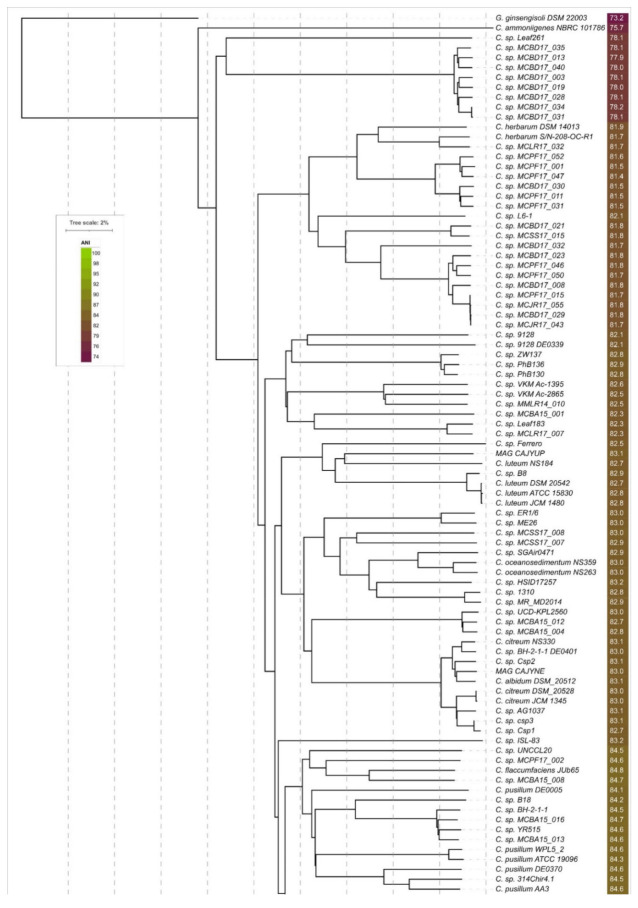

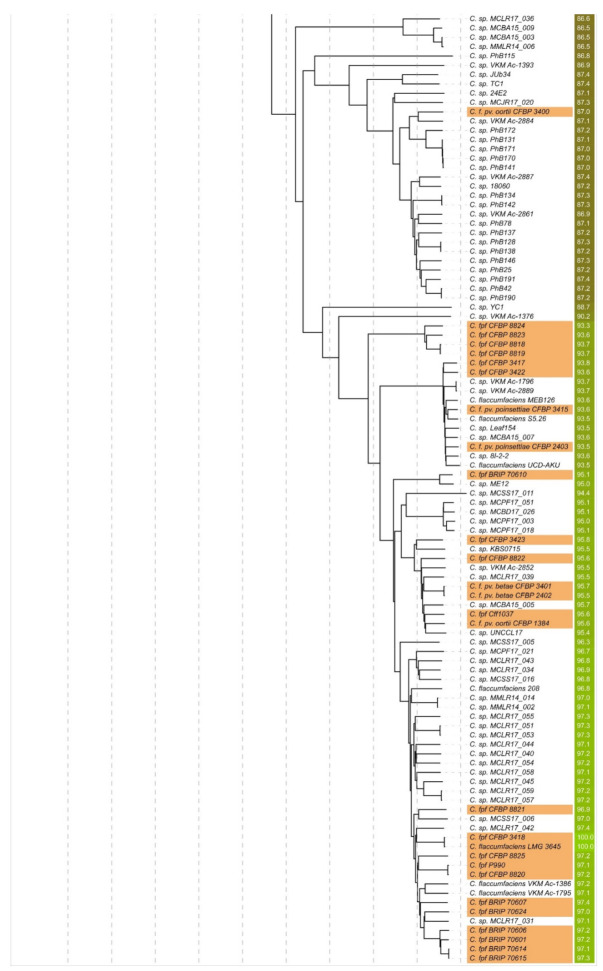
ANI tree plotted applying BioNJ clustering on 190 *Curtobacterium* genomes and *Gryllotalpicola ginnsengisoli* DSM 22003. The abbreviations are as follows: *C*.—*Curtobacterium*, *G*.—*Gryllotalpicola*, *C. f.*—*Curtobacterium flaccumfaciens*, and *C. fpf*—*flaccumfaciens* pv. *flaccumfaciens*. The *C. fpf* strains with confirmed pathogenicity are coloured yellow-orange. The scale bar shows 2% calculated genetic distance obtained by ANI calculations, and the trees were rooted to *Gryllotalpicola ginnsengisoli* DSM 22003. ANI values compared to the *C. fpf* CFBP 3418-type strain are shown to the right of the organism’s name and coloured according to a heat map scale.

In this study, the orthoANIu pipeline was used for the assessment of ANI. The advantage of orthoANIu is fast and accurate calculations due to the employment of the Usearch tool [68]. ANI values have been calculated for all 190 *Curtobacterium* genomes (Supplementary Figure S2) and used for BIONJ clustering (Figure 2). The dendrogram places most *C. flaccumfaciens* strains, including the type strains *C. fpf* CFBP 3418 and *C. f.* LMG 3645 and other strains with confirmed pathogenicity, into a distinct clade containing 69 strains with an ANI value above 92.5% compared to *C. flaccumfaciens*-type strains. The strain labelled as *C. f.* pv. *oortii* CFBP 3400, however, was placed in other clades distant from the other *C. flaccumfaciens* strains. These results indicate that the results of whole-genome based calculations do not match the present taxonomic classification of *C. flaccumfaciens* strains.

Interestingly, the ANI value of *C. ammoniigenes* NBRC 101786, compared to other *Curtobacterium* strains (75–76%, Supplementary Figure S1), was almost as low as the ANI value of *Gryllotalpicola ginnsengisoli* DSM 22003 (72–74%). According to the analysis of 3500 genomes representing type strains of species from >850 bacterial or archaeal genera [69], the ANI values of the prokaryotic genus demarcation boundaries have a mean of 73.98% (25% quartile, 70.85%; 75% quartile, 76.56%).

### 3.3. 16 S, 23S, and Concatenated Ribosomal RNA Genes Phylogeny

Genomic loci encoding ribosomal RNAs are often used for evolutionary phylogenetic analysis and species delineation [70,71,72]. As well as in other bacteria [73], the 16S rRNA and 23S rRNA genes of *Curtobacterium* are located in one operon, together with 5S rRNA, and are separated by internal transcribed spacer (ITS) regions. The majority of 16 complete *Curtobacterium* genomes contain three rRNA operons, and several complete genomes contain four copies of rRNA operons. In the latter case, two operons are located next to each other and are separated by the 1300–2200 base pairs (bp) region and are located in one direction. The average length of *Curtobacterium* 16S rRNA is about 1530 bases, and the average length of 23S rRNA is about 3120 nucleotides.

The copies of rRNA genes belonging to the same genome are often not identical. To conduct the phylogenetic analysis, the rRNA gene, which has an identical copy (copies), was chosen. If all the sequences were different and shared the same, or the adjacent, clade, the sequences with the least sum of branch lengths to the root of draft trees were chosen for the final tree.

The 16S sequence of *C. fpf* CFBP 3423 was found to be identical to *Moraxella osloensis* YV1 16S rRNA, which could have been the result of sample contamination and assembly error. Furthermore, this gene was not located in a common operon, together with the 23S and 5S rRNA genes. One of the 23S sequences of *C.* sp. HSID17257 was 100% identical to a *Cryptococcus neoformans* complex 25S rRNA and showed a high level of similarity with rRNA genes found in plasmids (*Actinomyces oris* strain FDAARGOS_1051 plasmid unnamed, *Enterobacter* sp. T2 plasmid unnamed, and *Acinetobacter baumannii* VB2139 plasmid pVB2139_3) and several bacteria.

The results of phylogenetic analysis of 16S rRNA genes belonging to 185 *Curtobacterium* strains (Supplementary Figures S2 and S3) raised doubts as to the employment of 16S phylogeny for *Curtobacterium* species delineation. A 16S maximum likelihood phylogeny could not distinguish the clades of *Curtobacterium* strains reliably—the bootstrap supporting values were often significantly lower than 50%. The use of different phylogeny inferring methods and nucleotide substitution models (RAxML, MrBayes, MEGA, TN93, GTR, TN93 + G + I, GTR + G + I, etc.) did not improve the robustness of inference and resulted in a similar consensus tree topology. This can be due to the low difference between the 16S rRNA sequences—the pairwise identity (the percentage of pairwise residues that are identical in the alignment, including gap versus non-gap residues but excluding gap versus gap residues) of the 16S rRNA alignment was as high as 99.4%.

The phylogenetic analysis of 23S rRNA genes belonging to 178 *Curtobacterium* strains (Supplementary Figures S4 and S5) seems to have been more informative, due to a bigger difference between the sequences (the pairwise identity of the 23S rRNA alignment was as high as 96.8%). The supporting bootstrap values were higher than in the case of 16S trees, and the composition of the clades revealed was often similar to the composition of ANI clusters (Figure 2). It seems, however, that the 23S was not able to describe the entire complexity of taxonomic and evolutionary relations within the *Curtobacterium* genus. The concatenated 16S and 23S rRNA genes’ phylogeny (Supplementary Figures S6 and S7) did not provide any improvement compared to the phylogeny of the single genes.

### 3.4. gyrB, parE, rpoA, rpoB, and Concatenated Genes Phylogeny

Conservative genes coding for proteins, mainly related to DNA processing (i.e., replication and transcription) and their concatenation, can be efficiently used for high-resolution phylogenetic analysis [74,75,76,77,78,79]. In this study, the nucleotide sequences of the following genes and their concatenation were used: gyrase subunit B (*gyrB*), topoisomerase IV subunit B (*parE*) evolutionarily related to gyrase B, DNA-directed RNA polymerase subunit α (*rpoA*), and DNA-directed RNA polymerase subunit β (*rpoB*). All the genes were found in single copies in almost all genome assemblies.

The phylogenetic trees of all the genes listed above (Supplementary Figures S8–S15) featured the overall resolution and bootstrap supporting values that were significantly higher than those in the rRNA phylogenetic trees. The composition of the revealed clades was close to the corresponding clades of the ANI tree, supporting the suggestion of the rarity of horizontal exchange events associated with the genes analysed. The gene *parE* encoding topoisomerase IV subunit B demonstrated good phylogenetic potential resolving the tree topology slightly better, and with a little better bootstrap support, than the *gyrB*, *rpoA*, and *rpoB* analyses, presumably because of the greater divergence of *parE*. The pairwise identities of the alignments were 91.4% for *gyrB* (189 sequences), 89.2% for *parE* (189 sequences), 96.6% for *rpoA* (189 sequences), and 95.7% for *rpoB* (188 sequences). It appears that a low number of variable sites hampers the fidelity and informativeness of the phylogeny.

The multilocus phylogenetic analysis (MLPA) (Supplementary Figures S16 and S17) employed the concatenated alignments of *gyrB*, *parE*, *rpoA*, and *rpoB* and demonstrated a higher resolution and better bootstrap support than the single genes phylogenies and, moreover, the rRNA phylogenies. The topologies of the MLPA trees and the compositions of the clades supported with high bootstrap values were close to those of the ANI tree (Figure 2). This concatenated tree did not, however, properly resolve the branches that were close to the root of the tree.

### 3.5. Ribosomal Proteins Phylogeny

The phylogenetic analysis based on the amino acid and nucleic acid sequences of ribosomal proteins (r-proteins) was shown to contain a reliable phylogenetic signal at a wide range of taxonomic depths, which was not significantly affected by mutational saturation or lateral gene transfer [80,81,82,83]. The phylogenetic studies made it possible to efficiently reveal the evolutionary and taxonomic relations between both distant, and closely related, organisms [84,85,86,87].

In this research, the concatenated nucleotide sequences of r-proteins extracted from reannotated genomes were used. The list of r-proteins genes used for concatenation comprised 46 genes: *rplA-rplF*, *rplI-rplP*, *rplR-rplY*, *rpmA*, *rpmB*, *rpmD*, *rpmE2*, *rpmG2*, *rpmH-rpmJ*, *rpsA-rpsD*, *rpsG-rpsO*, *rpsQ*, *rpsS*, and *rpsT*. The total length of the concatenated alignment was 21,202 bases; the pairwise identity was 92.4%.

**Figure 3:**
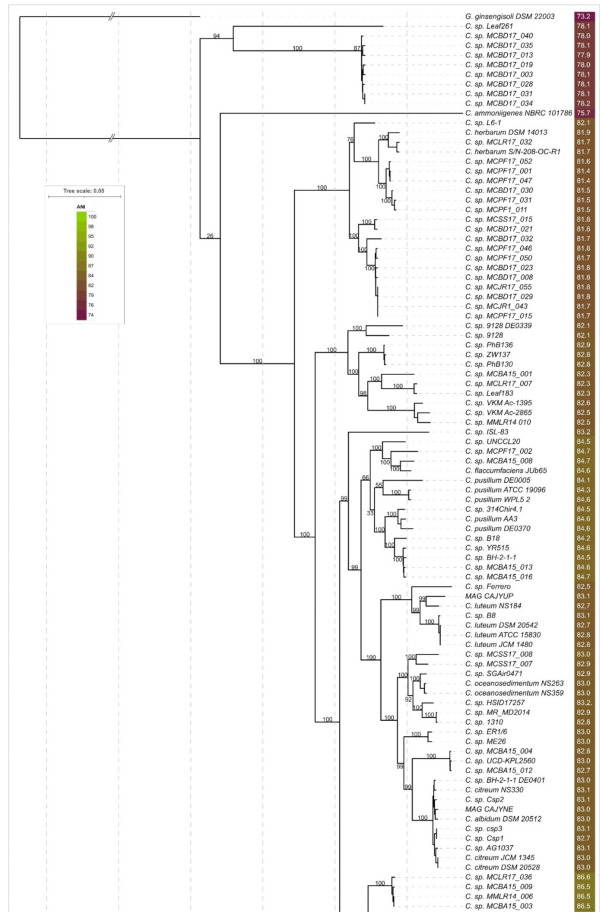

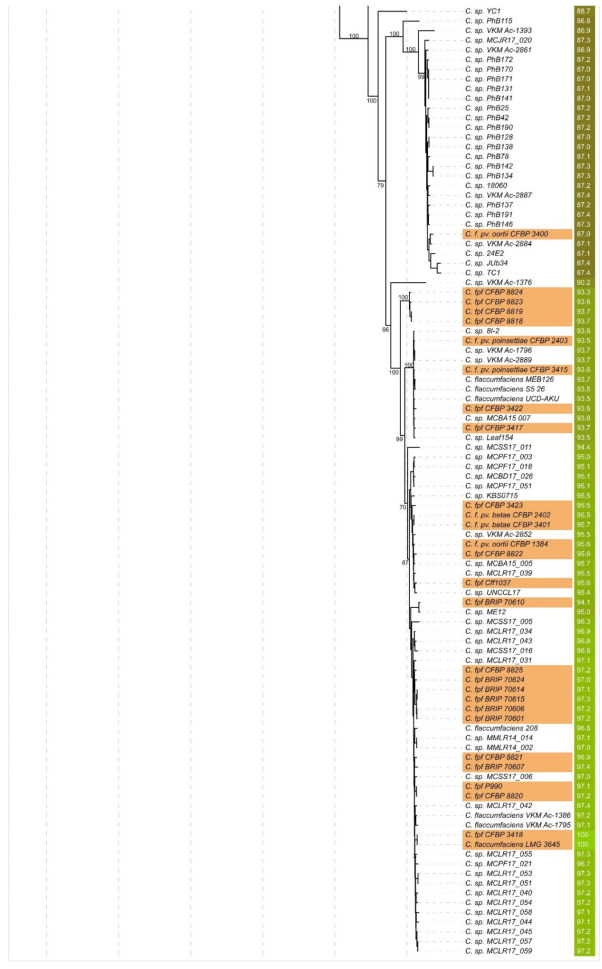
Best-scoring phylogenetic trees obtained with RAxML using concatenated nucleotide sequences’ alignments of ribosomal proteins extracted from 190 *Curtobacterium* genomes and *Gryllotalpicola ginnsengisoli* DSM 22003. The abbreviations are as follows: *C*.—*Curtobacterium*, *G*.—*Gryllotalpicola*, *C. f.*—*Curtobacterium flaccumfaciens*, and *C. fpf*—*flaccumfaciens* pv. *flaccumfaciens*. The *C. fpf* strains with confirmed pathogenicity are coloured yellow-orange. ANI values compared to the *C. fpf CFBP 3418*-type strain are shown to the right of the organism’s name and coloured according to a heat map scale. Bootstrap support values are shown near the branches of the rectangular tree as a percentage of 1000 replicates. The scale bar shows 0.05 estimated substitutions per site and the tree was rooted to *Gryllotalpicola ginnsengisoli* DSM 22003.

The resulting best-scoring phylogenetic tree obtained with RAxML (Figure 3) demonstrated bootstrap support that was significantly higher than that of the rRNA trees (Supplementary Figures S2–S7), four conservative genes’ trees, and four concatenated genes’ MLPA trees. The composition of clades, including the clade containing the strains classified as *C. flaccumfaciens*, was very close to the ANI tree (Figure 2).

### 3.6. Multigene-Based Phylogenomic Analysis

The employment of large-scale phylogenetic analyses involving many orthologous genes provides a substantial number of opportunities for the phylogeny and taxonomy of prokaryotes [60,88,89,90]. The multigene-based phylogenomic analysis was conducted using the core genes’ alignment, obtained with pangenome pipeline Panaroo, which uses a graph-based algorithm to share information between genomes, allowing improvements to annotation calls and the clustering of orthologues and paralogues within the pangenome [44]. The definition of a core (≥0.95) was applied, and 506 genes were found by the pipeline using all 190 reannotated genomes. The total length of the concatenated alignment was 502,785 bases, and the pairwise identity was 82.7%.

**Figure 4:**
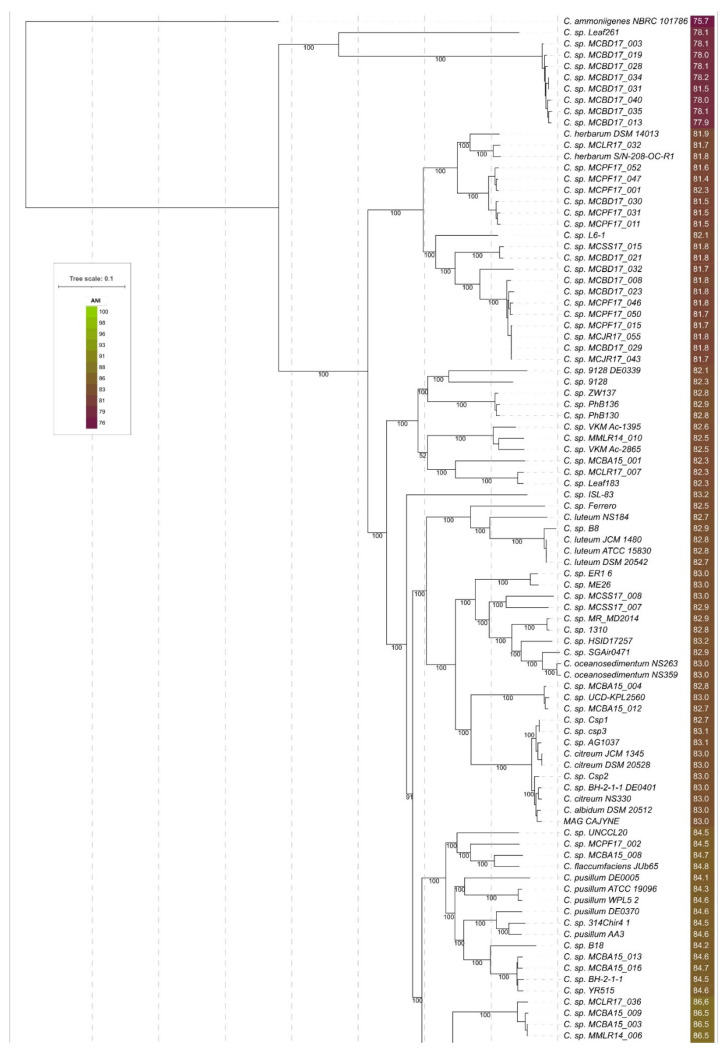

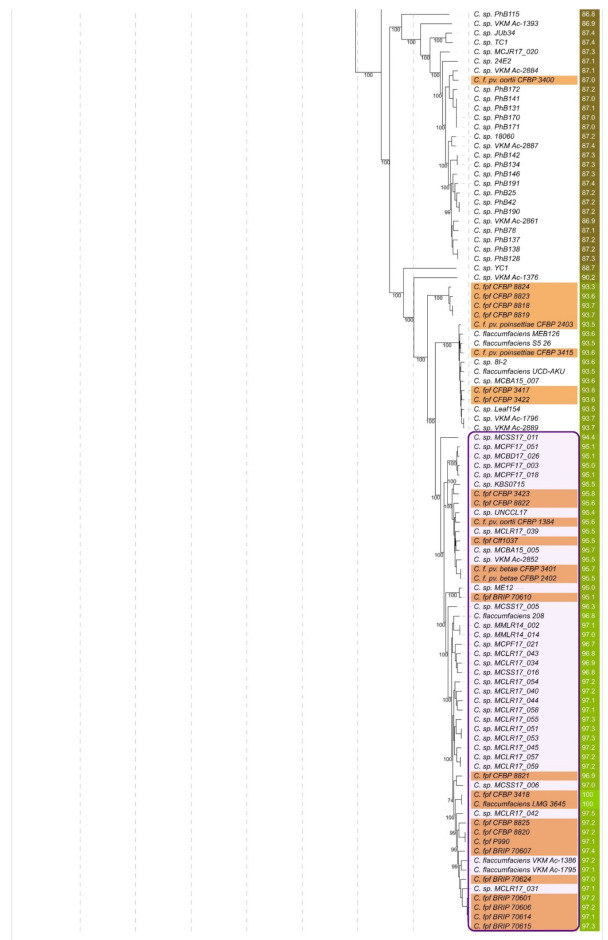
Best-scoring phylogenetic trees obtained with RAxML using 190 *Curtobacterium* genomes. The abbreviations are as follows: *C.*—*Curtobacterium*, *C. f.*—*Curtobacterium flaccumfaciens*, and *C. fpf*—*flaccumfaciens* pv*. flaccumfaciens*. The *C. fpf* strains with confirmed pathogenicity are coloured yellow-orange. The group of 53 strains outlined with violet constitutes a possible reclassified species of *C. flaccumfaciens*, based on the ANI and phylogeny results. ANI values compared to the *C. fpf CFBP* 3418-type strain are shown to the right of the organism’s name and coloured according to a heat map scale. Bootstrap support values are shown near the branches of the rectangular tree as a percentage of 1000 replicates. The scale bar shows 0.1 estimated substitutions per site, and the tree was rooted to *Curtobacterium ammoniigenes* NBRC 101786.

The ML best-scoring phylogenetic tree is shown in Figure 4. The tree demonstrates high bootstrap support and a topology that is similar to the topologies of ANI and r-protein trees. The compositions of the clades within the range of 95% ANI species cut-off are identical to those of the ANI tree (Figure 2).

### 3.7. Possible Taxonomy Revisions Based on ANI and Phylogenetic Analysis

Applying the criterion of full-genome similarity measured by ANI, which requires ≥95% identity and requirements of cladistics, which, in turn, demands the monophyleticity of taxa and using the results obtained by ANI calculations and phylogenetic analysis with high confidence, it is possible to propose the updates in the taxonomy of genus *Curtobacterium* that are shown in Table 2.

#### 3.7.1. Genomospecies 1. *Curtobacterium flaccumfaciens*

**Figure 5:**
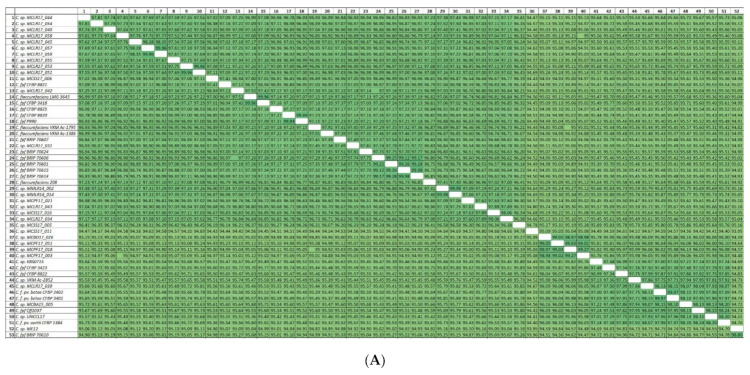

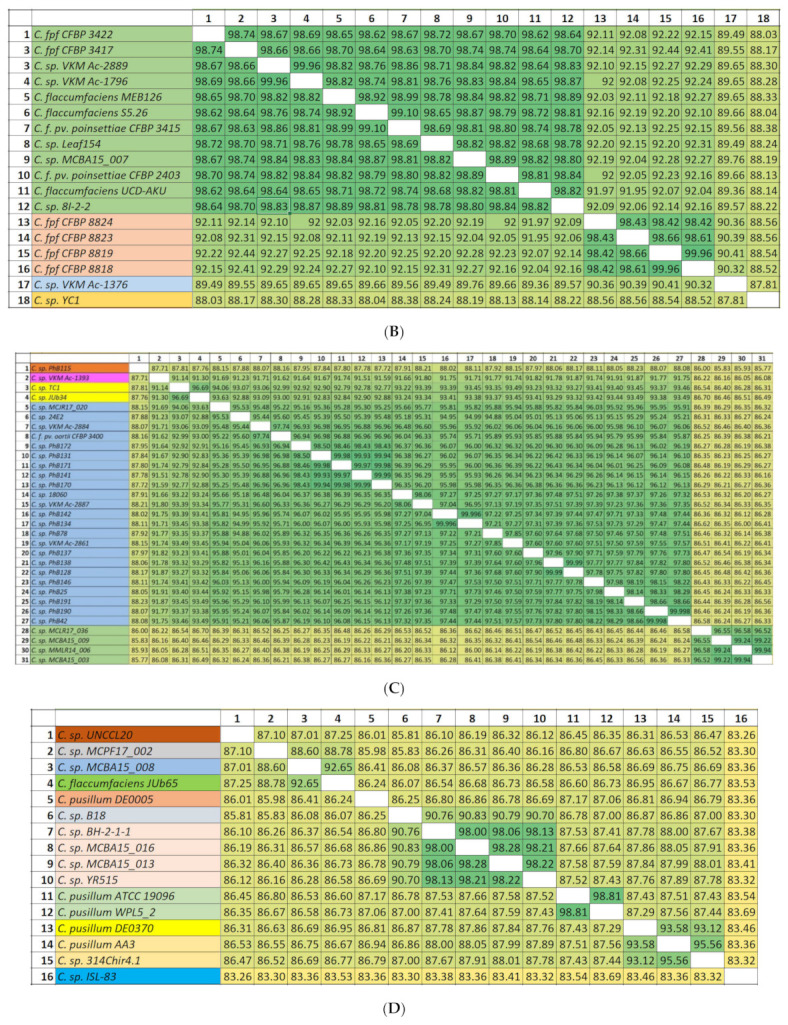

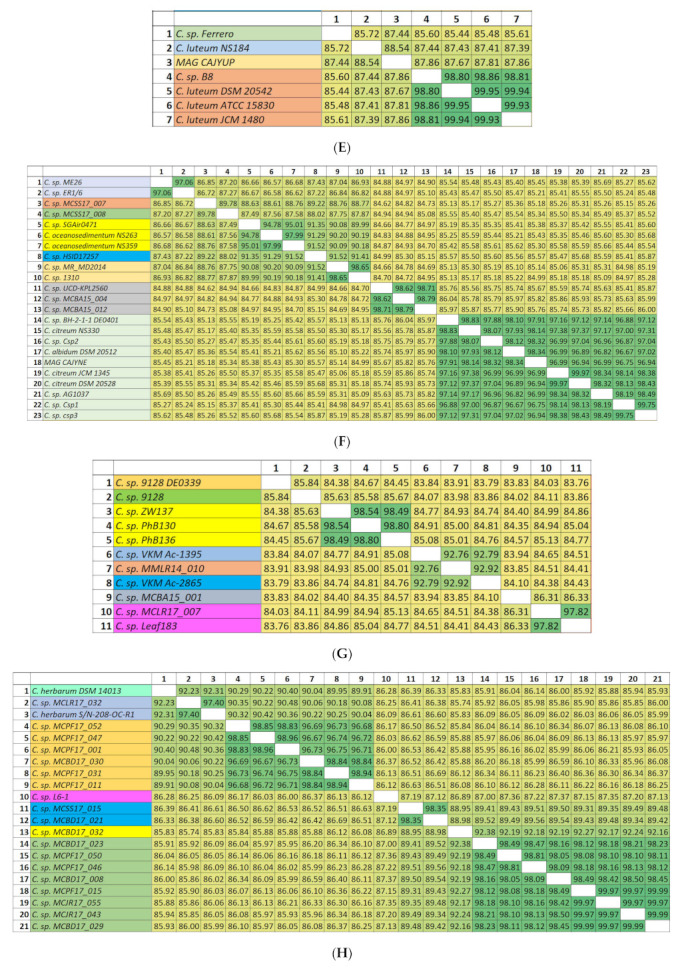

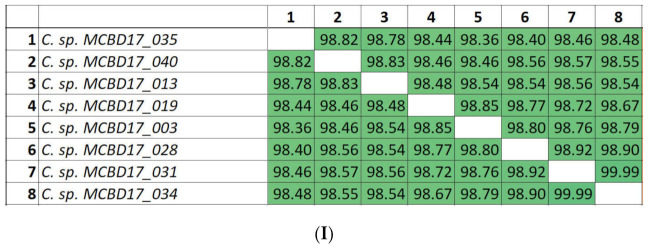
ANI matrix obtained by BioNJ clustering on 190 *Curtobacterium* genomes. (**A**) Group of strains proposed to be classified as *Curtobacterium flaccumfaciens*. (**B**–**I**) Clusters of strains containing proposed distinct genomospecies belonging to the genus of *Curtobacterium*. The strains which are coloured the same colour on the same image can be classified as representatives of the same genomospecies (Table 2). The abbreviations are as follows: *C*.—*Curtobacterium*, *C. f.*—*Curtobacterium flaccumfaciens*, and *C. fpf*—*flaccumfaciens* pv. *flaccumfaciens*.

Starting at the root of the core genome (Figure 4) or r-protein (Figure 3) trees, the monophyletic group of 53 strains, containing most phytopathogenic strains, can be classified as “*Curtobacterium flaccumfaciens*” (Figure 5A). The group includes the strains currently classified as *C. f.*, *C. fpf*, *C. f.* pv. *Betae*, *C. f.* pv. *Oortii*, and unclassified strains. The type strain can be replaced with another to maintain an ANI that is higher than the 95% threshold. This could be the strain CFBP 3423 or a different one. It appears that several subspecies might be established within this group.

#### 3.7.2. Genomospecies 2

The group of 12 strains comprising the strains currently classified as *C. f.*, *C. fpf*, *C. f.* pv. *Poinsettiae*, and unclassified strains (Figure 5B). To satisfy the requirement of ANI ≥ 95%, any of these strains can be suggested to be the type strain.

#### 3.7.3. Genomospecies 3–5

The group of four strains close to Species 2 phylogenetically and by ANI and comprising pathogenic strains *C. fpf* CFBP 8818, *C. fpf* CFBP 8819, *C. fpf* CFBP 8823, and *C. fpf* CFBP 8824 can be classified as a new genomospecies (Species 3), as well strains *C.* sp. VKM Ac-1376 (Species 4) and *C.* sp. YC1 (Species 5) (Figure 5B). The proposed genomospecies 1–5 include all the strains currently classified as *Curtobacterium flaccumfaciens*, except two.

#### 3.7.4. Genomospecies 6–10

The proposed genomospecies 6–10 constitute two related clades of phylogenetic trees (Figure 3 and Figure 4) and adjacent clusters of the ANI matrix (Figure 5C). These proposed species include one classified strain *C. f.* pv. *oortii* CFBP 3400 and 30 unclassified *Curtobacterium* strains.

#### 3.7.5. Genomospecies 11–21

The proposed genomospecies 11–20 belong to a single clade of phylogenetic trees (Figure 3 and Figure 4) and have ANI distance values that are large enough to distinguish them (Figure 5D). These species comprise five strains currently classified as *C. pusillum*, the strain labelled as *C. flaccumfaciens* JUb65, and ten unclassified *Curtobacterium* strains. Taking into account the genetic distances and phylogenetic data, the strains, currently classified as *C. pusillum*, should be assigned to three distinct species.

#### 3.7.6. Genomospecies 21–24

The phylogenetic trees’ clade and ANI matrix cluster (Figure 5E), comprising proposed genomospecies 21–24, contain seven strains, five of which are currently classified as *C. luteum*. One strain is unclassified, and one strain represents a metagenome assembly. It appears that the strains, currently classified as *C. luteum*, should be assigned to two different species.

#### 3.7.7. Genomospecies 26–33

Twenty-three strains, including the strains currently classified as *C. albidum*, *C. citreum*, *C. oceanosedimentum*, an unclassified draft genome, and a metagenome assembly, constitute a clade on phylogenetic trees (Figure 2, Figure 3 and Figure 4 and Figure 5F) and can be assigned to at least eight genomospecies. It is proposed that the strain *C.* sp. SGAir0471, as well as two strains labelled as *C. oceanosedimentum*, should be classified as *C. oceanosedimentum*, but the closeness of ANI to 95% requires further analysis of the taxonomic positions of *C.* sp. SGAir0471. It appears that the strains currently classified as *C. albidum* DSM 20512 and *C. citreum* should be assigned to one species.

#### 3.7.8. Genomospecies 34–41

The clade containing proposed genomospecies 34–41 comprises 11 unclassified strains (Figure 5G).

#### 3.7.9. Genomospecies 42–48

Twenty-three strains, including the strains currently classified as *C. herbarum* and 19 unclassified strains, can be assigned to seven species. It appears that, according to ANI and phylogenetic data, the strains, currently classified as *C. herbarum*, should be assigned to two distinct species.

#### 3.7.10. Genomospecies 49, 50

These two proposed genomospecies represent deeply rooted branches, which, nevertheless, may be assigned to the genus of *Curtobacterium*, according to the genomic data. The ANI values of the representatives of these species are about 78% compared to other proposed species. Genomospecies 49 contains eight unclassified strains (Figure 5I), and genomospecies 50 contains one unclassified strain.

#### 3.7.11. *C. ammoniigenes* NBRC 101786

According to the results of core genome phylogeny and ANI clustering, *C. ammoniigenes* NBRC 101786 represents the strain closest to the root of the trees. The ANI values of *C. ammoniigenes* NBRC 101786 are about 75–76% compared to other *Curtobacterium* strains. The taxonomic assignment of *C. ammoniigenes* should be the subject of a separate discussion. It might be possible to elevate this species to the level of a genus, taking into account biological and biochemical data.

### 3.8. Curtobacterium Plasmid pCff1 and Curtobacterium Plasmids

As of October 2021, there were ten *Curtobacterium* plasmid genomes in the NCBI Genome database (Table 3). They comprise a giant plasmid pCPAA3 featuring a genome of 567,298-bp size, five relatively large plasmids with genomes of 77–147 kbp, a medium-sized plasmid pTC5 of 42-kbp genome size, and two relatively small plasmids of 22–25 kbp. Interestingly, the two latter plasmids possess a GC content as low as about 32–35%, while the remaining plasmids are characterised by a GC content close to that of most *Curtobacterium* chromosomes (about 65–72%). The genomes of the plasmids with a low GC contain multiple repeats, CTTT, CCTTTT, and similar, with the overweight of the thymine residues.

The plasmids pCff1 and pCff113 attract special interest because of virulence proteins reported to be encoded in their genomes [23]. These plasmids have prominent within-genome similarities, with a linear plasmid, pCSL1, occurring in *Clavibacter sepedonicus* strain ATCC33113 [23].

**Figure 6:**
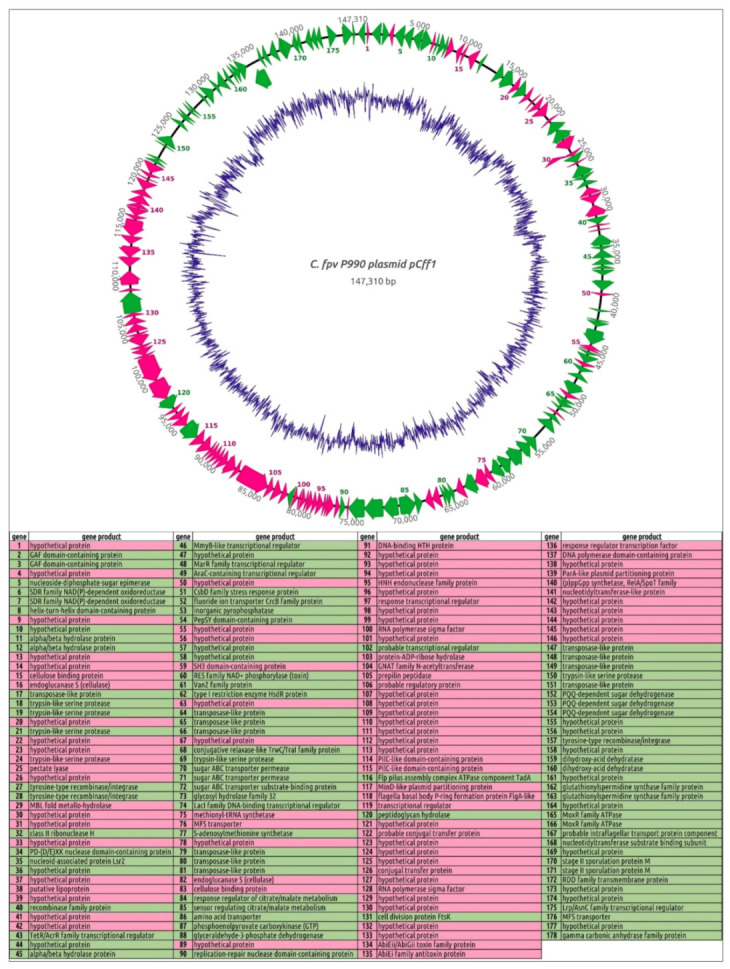
Circular genomic map and functional assignments of the *Curtobacterium flaccumfaciens* pv. *flaccumfaciens* P990 plasmid pCff1. In total, 178 protein-coding genes are shown as coloured blocks. The genes found in genomes of strains with confirmed pathogenicity in 50% or more of the total BLASTP search results on 190 *Curtobacterium* genomes are coloured green. The other genes are coloured light magenta. The direction of transcription is shown by arrows. The GC content of the genome sequence is indicated by the internal blue line.

The genome of pCff1 was reannotated using Prokka [25] and a thorough BLAST and HMM-HMM motif comparison involving NCBI and custom BLAST databases and databases offered by the HHpred [31], InterPro [33], and Phyre2 [32] servers (Figure 6). The reannotation predicted 178 open reading frames (ORFs). The list of genes that may be related to virulence includes a pectate lyase gene, two adjacent genes of cellulases and cellulose-binding proteins, six genes of trypsin-like serine proteases, and seven genes of putative hydrocarbon hydrolases. A BLAST search on these genes demonstrated the presence of their homologues in most, or all, pathogenic curtobacteria, while the majority of genes presumably not related with virulence were also found in the genomes of strains with pathogenicity not confirmed (Figure 6). Interestingly, close homologues of *Curtobacterium* trypsin proteases were found in pathovars of *Xanthomonas campestris* and *Xanthomonas citri*. Homologues of several supposed virulence genes were also found in phytopathogenic *Clavibacter*, *Dickeya*, and other phytopathogenic bacteria. Distant homologues of pectate lyase were found to be encoded by the genomes of phytopathogenic nematodes.

The pCff1 genome also contains the genes of conjugation apparatus, a toxin–antitoxin system, DNA repair and restriction, and several transposases.

**Figure 7:**
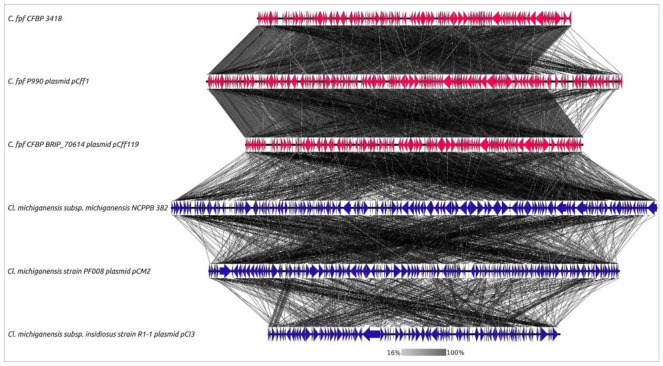
Genome sequence comparison among six *Curtobacterium* and *Clavibacter* plasmids and chromosome regions exhibiting co-linearity as detected by TBLASTX. The percentage of the sequence similarity is indicated by the intensity of the grey colour. Vertical blocks between analysed sequences indicate regions with at least 16% similarity. The abbreviations are as follows: *C*.—*Curtobacterium*, *Cl.*—*Clavibacter*, and *C. fpf*—*flaccumfaciens* pv. *flaccumfaciens*.

The sequence search on pCff1 demonstrated the presence of extended homologous regions in chromosomes of 13 *Curtobacterium* strains with confirmed pathogenicity, *Curtobacterium* sp. PhB130 and PhB136 chromosomes, and *Curtobacterium* plasmids pCff113 and pCff119 (Figure 7). In addition, the homology search and sequence alignments demonstrated the partial collinearity of pCff1 with *Clavibacter* plasmids pCI3 (genome length 80,801 bp), pCM2 (133,237 bp), and pVQ28-1 (89,287 bp).

### 3.9. pCff1 Putative DNA Polymerase Analysis

HMM–HMM motif comparison found meaningful similarities between gene product 137 of plasmid pCff1 and DNA polymerases of *Bacillus* phage φ29 [91] (HHpred [31] probability: 98.47%, E-value: 3.1 × 10^−5^); an unusual lemon-shaped archaeal virus *Salterprovirus* His1 (HHpred probability: 98.32%, E-value: 1.4 × 10^−4^); *Streptococcus* phage Cp-1 (HHpred probability: 98.19%, E-value: 2.6 × 10^−4^); human adenovirus C (HHpred probability: 97.91%, E-value: 1.3 × 10^−3^); and other bacterial, archaeal, and eukaryotic viruses. Significant similarities between phage φ29 and pCff1 gp137 were also shown by the I-TASSER homology modelling server [47] (TM-score 0.844, RMSD 1.35 Å), as well as more distant similarities with other viral DNA polymerases. However, the BLAST search did not reveal homologues of gp137 among the sequences, other than *Actinomycete* hypothetical proteins.

**Figure 8:**
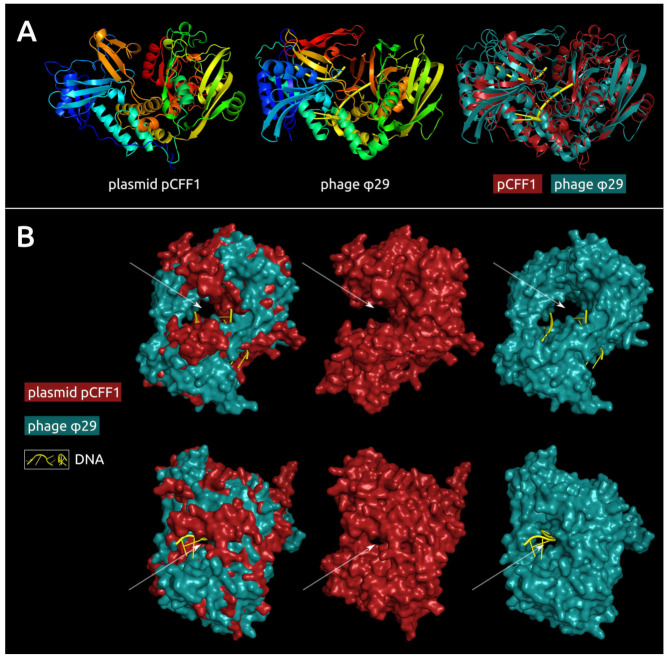
(**A**) Predicted structure of putative DNA polymerase from *C. fpf* P990 plasmid pCff1, the experimentally found structure of *Bacillus* phage φ29 DNA polymerase B complexed with DNA (PDB structure 2PY5) and their superimposition (RMSD 5.8 Å). The models are coloured based on a rainbow gradient scheme, where the N-terminus of the polypeptide chain is coloured blue, and the C-terminus is coloured red. (**B**) Superimposition of the predicted structure of putative DNA polymerase from plasmid pCff1, the predicted structure of pCff1 putative DNA polymerase and the experimentally found structure of φ29 DNA polymerase complexed with DNA using the protein surfaces. The arrows indicate the presence of similarly located cavities and tunnels in both proteins.

The structure of gp137 has been predicted using several approaches—homology modelling with Phyre2 and I-TASSER and deep learning algorithms implemented in AlphaFold2 [45] and RoseTTAFold [49]. According to the ModFOLD8 model quality assessment, the latter two demonstrated high predictive accuracy, which far exceeded that of homology modelling. The ModFOLD8 global model quality score was 0.1870 for Phyre2, 0.2188 for I-TASSER, 0.3804 for AlphaFold2, and 0.3720 for RoseTTAFold. The AlphaFold2 model refolded with ReFOLD3 [51] is shown in Figure 8. The final ModFOLD8 global model quality score was 0.3937, with high confidence and a *p*-value of 4.523 × 10^−3^; the predicted average residue error was 8.16 Å (Supplementary Figure S18).

A comparison of the predicted structure of plasmid pCff1 gp137 and phage φ29 demonstrates an overall similarity and the presence of similarly located cavities and tunnels in both proteins, which can be used in DNA binding and processing.

It is noteworthy that the genome of giant plasmids pCPAA3 also encodes for putative DNA polymerases. The BLAST search and HMM–HMM motif comparison predicted the presence of the genes of DNA polymerase III subunits α, ε, and κ. Furthermore, the pCPAA3 genome encodes a protein showing distant homology with the temperate *Siphoviridae Clavibacter* phage CN1A large subunit of terminase.

### 3.10. Curtobacterium PCR Diagnostics and Genus-Specific Primers

The unavailability of a selective medium for isolating *Curtobacterium* causes substantial difficulties for isolating new strains and studies of the biodiversity of *Curtobacterium* sp. The use of a semi-selective medium, in practice, still led to the isolation of visually similar bacterial strains from plants that did not belong to *Curtobacterium* sp. To simplify the selection procedure for field isolates of *Curtobacterium*, a set of genus-specific primers are proposed for the initial assessment of isolated samples using the qPCR method.

For the development of a diagnostic kit, a 23S rRNA region was chosen, which seemed to be conservative for *Curtobacterium* and differed from the outer groups. To amplify the selected genus-specific region, oligonucleotides were designed, the sequences of which are shown in Table 4.

To check the correctness of the identification with the designed primers, the assay was tested on the set of the strains indicated in Table S2.

The set of strains included 22 *Curtobacterium* strains purchased from the All-Russian Collection of Microorganisms (VKM) (No. 1–22) and eight *Curtobacterium* strains isolated by the authors from infected plants (No. 23–30), as well as 15 strains isolated according to the same protocol but which turned out to be representatives of other genera (No. 31–45). Additionally, a set of strains isolated from rotting plants was tested to confirm the absence of false-positive amplification with common phytopathogenic bacteria such as *Clavibacter, Pseudomonas, Xanthomonas, Pectobacterium, Dickeya*, and others (No. 46–81). To check the correctness of the amplification, the PCR product was sequenced for five random samples.

**Figure 9:**
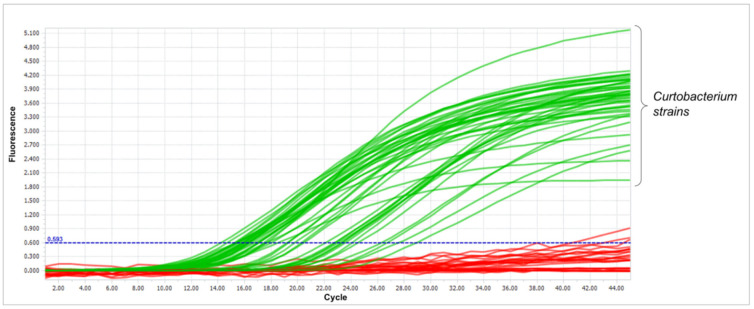
Amplification curves were obtained from the experiment to assess the selectivity of qPCR. The *Curtobacterium* strains are marked in green. Strains of other genera are marked in red.

The qPCR results are shown in Table S2, and an example of the amplification curves is shown in Figure 9. As can be seen from the data obtained, a positive signal was observed for all *Curtobacterium* strains, while no amplification was observed for non-*Curtobacterium* isolates.

According to the results obtained, amplification proceeded quite selectively, making it possible to differentiate *Curtobacterium* isolates from other microbiota found in infected plants, including closely related *Microbacteria*.

**Figure 10:**
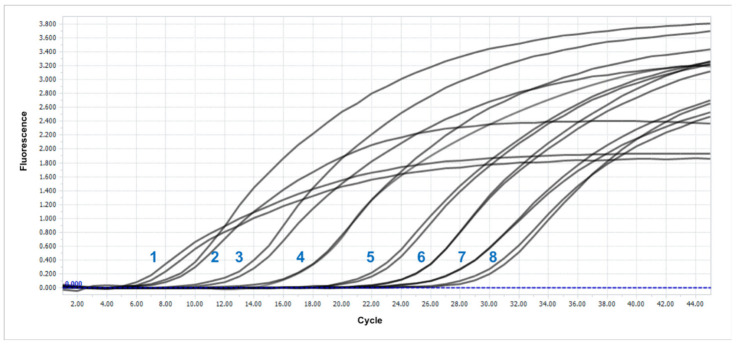
Amplification curves of tenfold dilutions of the test plasmid. The numbers represent the corresponding dilution shown in Table 5.

Additionally, for the qPCR detection system, the efficiency and sensitivity of the detection were evaluated. For greater accuracy in calculating the number of copies in the medium, a test plasmid was constructed. For this purpose, an amplified fragment of the target 23S rRNA region was ligated to the pDrive vector. With the resulting plasmid and the genomic DNA of the *C. flaccumfaciens* Ac-1923 (← DSM 20129) strain, serial tenfold dilutions and qPCR were conducted. The amplification curves for the plasmid experiment are shown in Figure 10.

**Figure 11:**
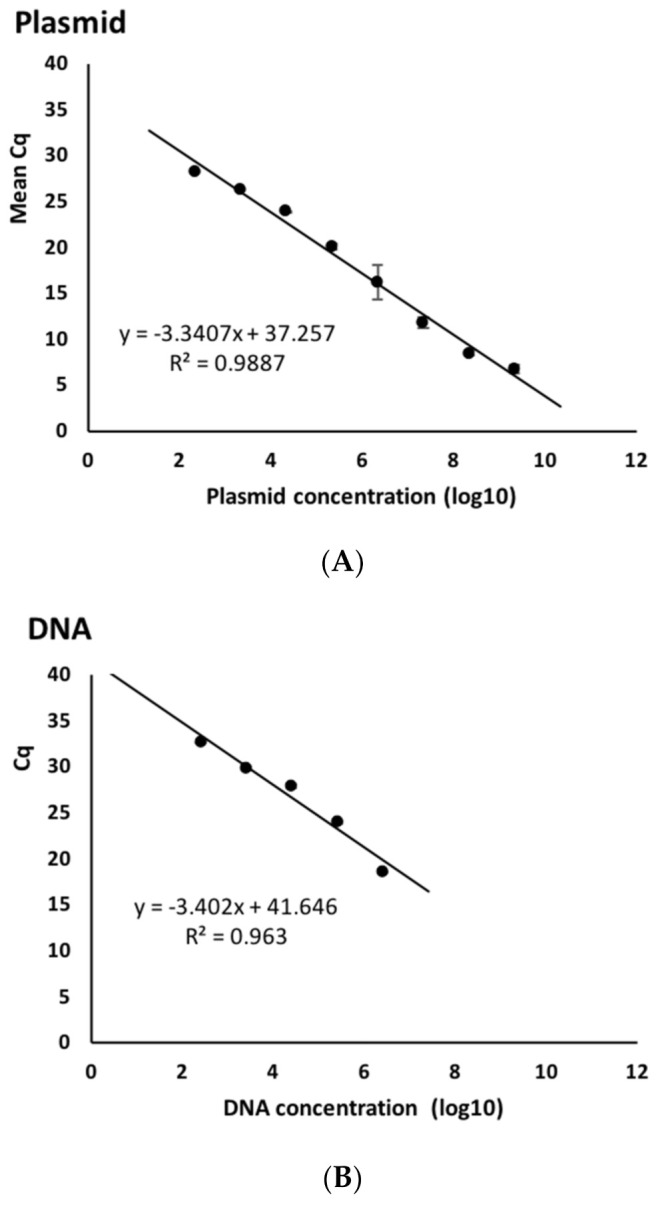
Standard curves obtained for dilutions of plasmid (**A**) and genomic (**B**) DNA.

The Cq values obtained made it possible to plot a graph of the dependence of the threshold cycle on the logarithm of the DNA concentration per reaction. The data obtained are presented in Table 5 and Figure 11. The figure shows that the standard curves obtained were linear, with slopes of 3.34 and 3.4 for the plasmid and genomic DNA, respectively. Thus, the PCR efficiency was 99.25 and 96.76%. The LoD in both cases was comparable and amounted to ≈10^3^ cfu/mL, which is a normal sensitivity for such detection systems.

## 4. Discussion

### 4.1. Challenges of Curtobacterium Taxonomic Classification

The genus *Curtobacterium* (family *Microbacteriaceae*) was defined by Yamada and Komagata in 1972 for a group of motile Brevibacteria [1]. It comprises a wide range of bacteria isolated from different environments and plants, including eight approved species:

1. *C. albidum* (Komagata and Iizuka 1964). Yamada and Komagata 1972 homotypic synonym: *Brevibacterium albidum* Komagata and Iizuka 1964 [1,92];
2. *C. ammoniigenes* (Aizawa et al. 2007 [93]);
3. *C. citreum* (Komagata and Iizuka 1964). Yamada and Komagata 1972 [1,92];
4. *C. flaccumfaciens* (Hedges 1922). Collins and Jones described this species as a plant pathogen with six recognised pathovars [3];
5. *C. herbarum* (Behrendt et al. 2002 [94]);
6. *C. luteum* (Komagata and Iizuka 1964). Yamada and Komagata 1972 [1,92];
7. *C. oceanosedimentum*—approved as a homotypic synonym: *Flavobacterium oceanosedimentum* Carty and Litchfield 1978 [95];
8. *C. pusillum* (Iizuka and Komagata 1965). Yamada and Komagata 1972 [1,92].

Other species have been proposed but not yet approved, such as *C. glycinis* from *Glycine max*, *C. gossypii* from *Gossypium hirsutum*, and *C. oryzae* sp. from *Oryza sativa* [96].

In the current work, 190 genomes of *Curtobacterium* spp. available at the NCBI Genome database were analysed using different bioinformatic methods. Deposited complete and draft genomes include 35 strains classified as *C. flaccumfaciens*, only 27 of which are identified as *C. flaccumfaciens* pathovars with confirmed virulence to a certain host plant. Seven genomes were classified as being representatives of other *Curtobacterium* species: *C. albidum*, *C. ammoniigenes*, *C. citreum*, *C. herbarum*, *C. luteum*, *C. oceanosedimentum*, and *C. pusillum* (Figure 1). Thus, 77.9% (148) of available genomes of *Curtobacterium* spp. have not yet been identified to a certain species. The relatively large number of sequenced genomes of *C. flaccumfaciens* (18.4%) can be explained by the status of *C. fpf* as an emerging and regulated plant pathogen that is spreading rapidly worldwide and that occurs worldwide in legume-producing countries.

The results of a phylogenetic analysis using ANI and conventional 16S rRNA and 23S rRNA genes showed limited applicability to the definition of species within the genus *Curtobacterium*. It is probable that this limitation is the major reason for the uncertain taxonomic positioning of 148 analysed genomes.

Using the sequences of concatenated conservative genes coding for DNA processing proteins (*gyrB*, *parE*, *rpoA*, and *rpoB*) produced phylogenetic trees with significantly higher resolution and bootstrap supporting values than the rRNA phylogenetic analysis. The topology of these trees (Supplementary Figures S8–S15) suggests the rare occurrence of horizontal exchange events among the chosen genes.

A phylogenetic analysis based on concatenated nucleotide sequences of 46 ribosomal proteins (r-proteins) extracted from reannotated genomes was used. The resulting best-scoring phylogenetic tree obtained with RAxML (Figure 4) demonstrated bootstrap support that was significantly higher than that of all other trees (Supplementary Figures S2–S7). The composition of the clades, including the clade containing the strains classified as *Curtobacterium flaccumfaciens*, was very close to that of the ANI tree (Figure 2).

Applying the criterion of full-genome similarity as measured by ANI, which requires ≥95% identity and requirements for the monophyleticity of taxa, and using the results obtained by ANI calculations and phylogenetic analysis with a high level of confidence, a more detailed taxonomy of genus *Curtobacterium* with 51 potential genomospecies can be proposed (Table 2). A group of 53 monophyletic strains can be assigned to the species *C. flaccumfaciens*, which can include both pathogenic and non-pathogenic strains. All results presented in this work indicate the necessity for taxonomic revisions within the genus of *Curtobacterium* and correlate with previous genomic studies.

Chen et al. [22] analysed 45 strains from the NCBI GenBank database designated as *Curtobacterium* spp. The ANI values in pairs of these strains varied from 75% to 99% and, using standard criteria based on ANI and dDDH [97], showed that only four strains belonged to *C. flaccumfaciens sensu stricto* among the evaluated genome sequences. The strains UNCCL17 and MCBA15-005 were phylogenetically closely related to *C. flaccumfaciens*, but it is likely that they belong to a novel species. According to Reference [22], *sensu stricto C. flaccumfaciens* strains were clustered in a monophyletic clade showing 97% ANI with one another.

MLSA based on housekeeping genes recA, gyrB, ppK, atpD, dnaK, and rpoB was used to investigate the phylogenetic relationships among 84 strains of C. flaccumfaciens, which were distributed among four pathovars (*C. flaccumfaciens* pv. *flaccumfaciens*, poinsettiae, oortii, and betae) and isolated from host plants over a period of 77 years from North America, South America, Europe, and Asia. The *C. flaccumfaciens* strains were grouped into three main clusters. Colony colour aside, the three clusters did not correlate with pathovar affiliation, isolation date, geographical location, or isolation host. Thus, the pv. *flaccumfaciens* strains, pathogenic on beans, were scattered among the three clusters as diverse as other Curtobacterium species. Strains from the same pathovars (hosts of isolation) were placed in different clusters, irrespective of their geographical origin [98].

A core genome phylogeny of 50 genomes [99] provided evidence that *Curtobacterium* spp. strains with glycosyl hydrolases (GHs) and, thus, with the potential for being degraders of cellulose and other polysaccharides, were not phylogenetically related to the type strain of *C. flaccumfaciens*, because they shared only 85% ANI. Furthermore, strains GD1, BH-2-1-1, MCBA15_013, and YR515 probably belong to separate species that are currently unnamed.

Chen et al. [22] evaluated 45 bacterial strains designated as *C. flaccumfaciens* in the literature and found the genetic diversity within the group to be greater than had so far been described. Only two strains, MMLR14-002 and MMLR14-014, were identified as being members of *C. flaccumfaciens*, while the remaining strains needed to be reclassified as novel taxa.

Recently, MLST, using the sequences of five housekeeping genes (i.e., *atpD*, *gyrB*, *ppk*, *recA*, and *rpoB*), revealed that the three strains, MCBA15-007, MEB126, and UCD-AKU, were phylogenetically closely related to the type strain of the poinsettia (*Euphorbia pulcherrima*) pathogen (pv. *poinsettiae*) ICMP 2566T, while the strains MCBA15-005 and UNCCL17 were phylogenetically closely related to the type strain of the sugar beet pathogen (pv. *betae*) ICMP 2594T [21]. The authors indicated that the plant pathogenic members of *C. flaccumfaciens* must be attributed to different species, reinforcing the need to reconsider the taxonomy of phytopathogenic members of the species. Thus, previous evaluations of *Curtobacterium* strains using genome sequences or MLST/MLSA have illustrated the presence of strain groups that are different enough from type strains of the eight validated species for new unnamed species to be claimed.

Jain et al. [62] computed the pairwise average nucleotide identity (ANI) of 91,761 microbial genomes and found that the ANI values calculated from the 8 billion comparisons showed a strong bimodal distribution concentrated at 83 and 95%, with a wide gap between these two peaks. The authors concluded that a clear genetic discontinuum and species boundary were evident from the unprecedented large-scale ANI analysis and claimed that the 95% ANI threshold represented an accurate threshold for demarcating almost all currently named prokaryotic species. Murray et al. [100] argued that the creation of a universal genetic boundary among the named species in the current NCBI taxonomy was questionable and that it resulted from substantially biased sampling in genome sequencing. They urged caution against being excessively confident in using 95% ANI for microbial species delineation, since the high benchmarks reported in the paper were inflated by the use of highly redundant genomes. Microbes occupying narrow ecological niches and with a limited dispersal rate (e.g., obligate intracellular bacteria) may develop genetic clusters. Free living microbes exploring different habitats are more likely to exhibit a genetic continuum. Selection is also unlikely to produce a universal genetic boundary, as microbial species are unique in nature, with each species subject to its own evolutionary and ecological forces [100].

In answer to the critics of the general concept of Jain et al. [62], the authors said that some bacterial populations “show large intrapopulation sequence diversity, probably due to unique ecological niche(s) they have occupied for long evolutionary times compared to other marine taxa (i.e., they lack direct competition), and thus this threshold is around 90–92% ANI for these populations. In contrast, several more recently emerged pathogens like *Bacillus anthracis* show limited intrapopulation species diversity (ANI values > 99%). Hence, the area of genetic discontinuity may vary, depending on the taxa considered and their unique ecophysiologic and evolutionary characteristics, and 95% ANI appears to be the genetic level that distinguishes most natural discrete populations and named species, but not necessarily all.”

It was concluded that, “for these reasons, taxon descriptions should not be based on a single metric or threshold but the careful investigation of ecological and functional data together with genetic relatedness (e.g., ANI values)” [101].

### 4.2. Curtobacterium Plasmids and Pathogenicity

The occurrence of pCff1-related plasmids in the pathogenic strains of *Curtobacterium* and *Clavibacter* strains appears to be related to their virulence. The important role of plasmids has been shown for many phytopathogenic bacteria [102,103,104,105]. The analysis of, and homology search on, the genes of those plasmids indicated their presence mainly in virulent strains with pathogenicity confirmed. The occurrence of putative virulence genes in strains with no confirmed pathogenicity may be explained by the lack of experimentation on their pathogenic behaviour, their past lifestyle, and other benefits from the presence of such genes. For example, the presence of enzymes that assist in the use of plant cell walls and polymers present in the integuments of hosts is related to both saprotrophic nutrition and pathogenic lifestyle [106,107]. However, if such enzymes are useful for saprophytes, they may be obligate for pathogens.

Discovery of the gene that encodes the protein structurally similar to viral DNA polymerase I (which belongs to family B DNA polymerases) raises important questions about the origin and evolution of pCff1-related plasmids. A plasmid-encoded DNA polymerase is an unusual event for bacteria and archaea, but several incidents, including actinomycetes, have been reported [108,109,110,111,112]. Plasmid DNA polymerase genes are not unusual for plasmids of fungal mitochondria [113,114,115,116,117,118], including the mitochondria of a phytopathogenic fungus *Claviceps purpurea* [119]. Additionally, DNA polymerases are encoded in some temperate bacteriophages like N15, which persist like a linear plasmid in a host cell [120]. It is noteworthy that mitochondrial plasmid and phage N15 polymerases appear to belong to family B DNA polymerases.

The homologues of pCff1 putative DNA polymerase have been detected only in chromosomal and plasmid genomes of actinomycetes. The relatedness of pCff1 putative DNA polymerase with the DNA polymerases of bacteriophages and archaeal and eukaryotic viruses have been revealed with the HMM–HMM motif comparison and structure modelling. These analyses have demonstrated that pCff1 putative DNA polymerase is more similar to the phage DNA polymerase. It is possible to hypothesise about the comparatively ancient origin and divergence of this protein. It is necessary to study the functioning of this enzyme experimentally. It might be suggested that the pCff1 putative DNA polymerase I could participate in the initial stages of replication, as has been reported in the context of some other plasmids [121,122,123].

### 4.3. Curtobacterium spp. PCR Diagnostics

The current study has seen, for the first time, the development of a genus-specific primer set for the diagnosis of *Curtobacterium*. The set makes it possible to differentiate *Curtobacterium* from representatives of other microbiota of an infected plant, which is an urgent applied problem. Primers for the species-specific detection of Cff have previously been described in the literature [124], but there have been no kits for genus-specific diagnostics. The high level of efficiency of PCR with the test system developed and with a sufficiently low detection limit have been shown experimentally. The values are comparable to those for similar qPCR systems for other genera of phytopathogens [125].

The resulting test system can become a valuable and easy-to-use tool for rapidly assessing the strains isolated from a diseased plant. In addition, the sensitivity of detection should allow the use of this method for assessing plant extracts for the presence of a pathogen, even in asymptomatic plants.

## 5. Conclusions

ANI comparisons and the phylogenetic analysis of ribosomal and core proteins testify to the necessity for global taxonomic revisions within the genus of *Curtobacterium*. Based on these data, it is possible to discuss the delineation of up to several dozen genomospecies. A monophyletic group of 53 strains can be assigned to the species of *Curtobacterium flaccumfaciens*, which can include both pathogenic and non-pathogenic strains. The pathogenicity of *Curtobacterium* and other *Microbacteriaceae* can be related to a group of plasmids carrying virulence factors and featuring the presence of a gene distantly related to viral DNA polymerase I. The recent increase in genomic data challenges widespread diagnostic methods. The presented genus-specific PCR diagnostic kit developed and tested in the present work may serve as a good complementary tool for further studies of *Curtobacterium* strains

## Figures and Tables

**Table 1:** *Curtobacterium flaccumfaciens* strains with confirmed pathogenicity.

NCBI Accession	Strain	Isolation Source	Source
JABMCF	*C. f. strain* LMG 3645 = CFBP3418	beans	1957 Klement, Z.
JAHEXD	*C. f.* pv. *betae strain* CFBP 2402	beet	1955 Keyworth, W.G.
JAHEWW	*C. f.* pv. *betae strain* CFBP 3401	beet	Keyworth, W.
JAFJLX	*C. f.* pv. *flaccumfaciens strain* BRIP 70601	mungbean	Vaghefi, N.
CP074439	*C. f.* pv. *flaccumfaciens strain* BRIP:70606	mungbean	-
JAFJLW	*C. f.* pv. *flaccumfaciens strain* BRIP 70607	mungbean	-
JAFJLV	*C. f.* pv. *flaccumfaciens strain* BRIP 70610	mungbean	-
CP071883	*C. f.* pv. *flaccumfaciens strain* BRIP 70614	mungbean	-
JAFJLU	*C. f.* pv. *flaccumfaciens strain* BRIP 70615	mungbean	-
JAFJLT	*C. f.* pv. *flaccumfaciens strain* BRIP 70624	mungbean	-
PUEZ	*C. f.* pv. *flaccumfaciens strain* CFBP3418	beans	1957 Klement, Z.
JAHEWX	*C. f.* pv. *flaccumfaciens strain* CFBP 3417	beans	1958 Lelliott, R.A
JAHEWY	*C. f.* pv. *flaccumfaciens strain* CFBP 3422	beans	1956 Schuster, M.L.
JAHEWZ	*C. f.* pv. *flaccumfaciens strain* CFBP 3423	beans	1957 Schuster, M.L.
JAHEWT	*C. f.* pv. *flaccumfaciens strain* CFBP 8818	tomato	2015 Osdaghi, E.
JAHEWS	*C. f.* pv. *flaccumfaciens strain* CFBP 8819	tomato	-
JAHEWR	*C. f.* pv. *flaccumfaciens strain* CFBP 8820	tomato	-
JAHEWQ	*C. f.* pv. *flaccumfaciens strain* CFBP 8821	tomato	-
JAHEWP	*C. f.* pv. *flaccumfaciens strain* CFBP 8822	tomato	-
JAHEWO	*C. f.* pv. *flaccumfaciens strain* CFBP 8823	tomato	2015 Osdaghi, E.
JAHEWN	*C. f.* pv. *flaccumfaciens strain* CFBP 8824	tomato	-
JAHEWM	*C. f.* pv. *flaccumfaciens strain* CFBP 8825	tomato	-
CP041259	*C. f.* pv. *flaccumfaciens strain* Cff1037	beans	2015 Osdaghi, E.
CP045287	*C. f.* pv. *flaccumfaciens strain* P990	dry beans	2015 Osdaghi, E.
JAHEXC	*C. f.* pv. *oortii strain* CFBP 1384	tulip	1967 Barendsen, H.
JAHEXA	*C. f.* pv. *oortii strain* CFBP 3400	arum lily	1990 Janse, J.D.
JAHEXB	*C. f.* pv. *poinsettiae strain* CFBP 2403	euphorbia	Starr, M.P.
JAHEWU	*C. f.* pv. *poinsettiae strain* CFBP 3415	euphorbia	Dye, D.

**Table 2:** Suggested *Curtobacterium* species based on the genomic data.

Species	Strains
Genomospecies 1. *C. flaccumfaciens*	*C. f.* pv. *betae* CFBP 2402, *C. f.* pv. *betae* CFBP 3401, *C. f.* pv. *oortii* CFBP 1384, *C. flaccumfaciens* 208, *C. flaccumfaciens* LMG 3645, *C. flaccumfaciens* VKM Ac-1386, *C. flaccumfaciens* VKM Ac-1795, *C. fpf* BRIP 70601, *C. fpf* BRIP 70606, *C. fpf* BRIP 70607, *C. fpf* BRIP 70610, *C. fpf* BRIP 70614, *C. fpf* BRIP 70615, *C. fpf* BRIP 70624, *C. fpf* CFBP 3418, *C. fpf* CFBP 3423, *C. fpf* CFBP 8820, *C. fpf* CFBP 8821, *C. fpf* CFBP 8822, *C. fpf* CFBP 8825, *C. fpf* Cff1037, *C. fpf* P990, *C.* sp. KBS0715, *C.* sp. MCBA15_005, *C.* sp. MCBD17_026, *C.* sp. MCLR17_031, *C.* sp. MCLR17_034, *C.* sp. MCLR17_039, *C.* sp. MCLR17_040, *C.* sp. MCLR17_042, *C.* sp. MCLR17_043, *C.* sp. MCLR17_044, *C.* sp. MCLR17_045, *C.* sp. MCLR17_051, *C.* sp. MCLR17_053, *C.* sp. MCLR17_054, *C.* sp. MCLR17_055, *C.* sp. MCLR17_057, *C.* sp. MCLR17_058, *C.* sp. MCLR17_059, *C.* sp. MCPF17_003, *C.* sp. MCPF17_018, *C.* sp. MCPF17_021, *C.* sp. MCPF17_051, *C.* sp. MCSS17_005, *C.* sp. MCSS17_006, *C.* sp. MCSS17_011, *C.* sp. MCSS17_016, *C.* sp. ME12, *C.* sp. MMLR14_002, *C.* sp. MMLR14_014, *C.* sp. UNCCL17, *C.* sp. VKM Ac-2852
Genomospecies 2	*C. f.* pv. *poinsettiae* CFBP 2403, *C. f.* pv. *poinsettiae* CFBP 3415, *C. flaccumfaciens* MEB126, *C. flaccumfaciens* S5.26, *C. flaccumfaciens* UCD-AKU, *C. fpf* CFBP 3417, *C. fpf* CFBP 3422, *C.* sp. 8I-2-2, *C.* sp. Leaf154, *C.* sp. MCBA15_007, *C.* sp. VKM Ac-1796, *C.* sp. VKM Ac-2889
Genomospecies 3	*C. fpf* CFBP 8818, *C. fpf* CFBP 8819, *C. fpf* CFBP 8823, *C. fpf* CFBP 8824
Genomospecies 4	*C.* sp. VKM Ac-1376
Genomospecies 5	*C.* sp. YC1
Genomospecies 6	*C.* sp. PhB115
Genomospecies 7	*C.* sp. VKM Ac-1393
Genomospecies 8	*C.* sp. JUb34, *C.* sp. TC1
Genomospecies 9	*C. f.* pv. *oortii* CFBP 3400, *C.* sp. 18060, *C.* sp. 24E2, *C.* sp. MCJR17_020, *C.* sp. PhB128, *C.* sp. PhB131, *C.* sp. PhB134, *C.* sp. PhB137, *C.* sp. PhB138, *C.* sp. PhB141, *C.* sp. PhB142, *C.* sp. PhB146, *C.* sp. PhB170, *C.* sp. PhB171, *C.* sp. PhB172, *C.* sp. PhB190, *C.* sp. PhB191, *C.* sp. PhB25, *C.* sp. PhB42, *C.* sp. PhB78, *C.* sp. VKM Ac-2861, *C.* sp. VKM Ac-2884, *C.* sp. VKM Ac-2887
Genomospecies 10	*C.* sp. MCBA15_003, *C.* sp. MCBA15_009, *C.* sp. MCLR17_036, *C.* sp. MMLR14_006
Genomospecies 11	*C.* sp. UNCCL20
Genomospecies 12	*C.* sp. MCPF17_002
Genomospecies 13	*C.* sp. MCBA15_008
Genomospecies 14	*C. flaccumfaciens* JUb65
Genomospecies 15	*C. pusillum* DE0005
Genomospecies 16	*C.* sp. B18
Genomospecies 17	*C.* sp. BH-2-1-1, *C.* sp. MCBA15_013, *C.* sp. MCBA15_016, *C.* sp. YR515
Genomospecies 18	*C. pusillum* ATCC 19096, *C. pusillum* WPL5_2
Genomospecies 19	*C. pusillum* DE0370
Genomospecies 20	*C. pusillum* AA3, *C.* sp. 314Chir4.1
Genomospecies21	*C.* sp. ISL-83
Genomospecies 22	*C.* sp. Ferrero
Genomospecies 23	*C. luteum* NS184
Genomospecies 24	Metagenome assembly accession CAJYUP
Genomospecies 25	*C. luteum* ATCC 15830, *C. luteum* DSM 20542, *C. luteum* JCM 1480, *C.* sp. B8
Genomospecies 26	*C.* sp. ER1/6, *C.* sp. ME26
Genomospecies 27	*C.* sp. MCSS17_007
Genomospecies 28	*C.* sp. MCSS17_008
Genomospecies 29	*C. oceanosedimentum* NS263, *C. oceanosedimentum NS359*, *C.* sp. SGAir0471
Genomospecies 30	*C.* sp. HSID17257
Genomospecies 31	*C.* sp. 1310, *C.* sp. MR_MD2014
Genomospecies 32	*C.* sp. MCBA15_004, *C.* sp. MCBA15_012, *C.* sp. UCD-KPL2560
Genomospecies 33	*C. albidum* DSM 20512, *C. citreum* DSM 20528, *C. citreum* JCM 1345, *C. citreum* NS330, *C.* sp. AG1037, *C.* sp. BH-2-1-1 DE0401, *C.* sp. Csp1, *C.* sp. Csp2, *C.* sp. csp3, Metagenome assembly accession CAJYNE
Genomospecies 34	*C.* sp. 9128 DE0339
Genomospecies 35	*C.* sp. 9128
Genomospecies36	*C.* sp. PhB130, *C.* sp. PhB136, *C.* sp. ZW137
Genomospecies 37	*C.* sp. VKM Ac-1395
Genomospecies 38	*C.* sp. MMLR14_010
Genomospecies 39	*C.* sp. VKM Ac-2865
Genomospecies 40	*C.* sp. MCBA15_001
Genomospecies 41	*C.* sp. Leaf183, *C.* sp. MCLR17_007
Genomospecies 42	*C. herbarum* DSM 14013
Genomospecies 43	*C. herbarum* S/N-208-OC-R1, *C.* sp. MCLR17_032
Genomospecies 44	*C.* sp. MCBD17_030, *C.* sp. MCPF17_001, *C.* sp. MCPF17_011, *C.* sp. MCPF17_031, *C.* sp. MCPF17_047, *C.* sp. MCPF17_052
Genomospecies 45	*C.* sp. L6-1
Genomospecies 46	*C.* sp. MCBD17_021, *C.* sp. MCSS17_015
Genomospecies 47	*C.* sp. MCBD17_032
Genomospecies 48	*C.* sp. MCBD17_008, *C.* sp. MCBD17_023, *C.* sp. MCBD17_029, *C.* sp. MCJR17_043, *C.* sp. MCJR17_055, *C.* sp. MCPF17_015, *C.* sp. MCPF17_046, *C.* sp. MCPF17_050
Genomospecies 49	*C.* sp. MCBD17_003, *C.* sp. MCBD17_013, *C.* sp. MCBD17_019, *C.* sp. MCBD17_028, *C.* sp. MCBD17_031, *C.* sp. MCBD17_034, *C.* sp. MCBD17_035, *C.* sp. MCBD17_040
Genomospecies 50	*C.* sp. Leaf261
Genomospecies 51/Genus	*C. ammoniigenes* NBRC 101786

**Table 3:** *Curtobacterium* plasmid genomes deposited in the NCBI Genome database as of October 2021.

NCBI Accession	Plasmid	% GC	Sequence Length	Topology
CP018784	*C. pusillum* strain AA3 plasmid pCPAA3	66.7%	567,298	circular
CP041260	*C. fpf* strain Cff1037 plasmid pCff113	66.1%	113,440	linear
CP045288	*C. fpf* strain P990 plasmid pCff1	66.1%	147,310	circular
CP045289	*C. fpf* strain P990 plasmid pCff2	32.3%	25,142	circular
CP045290	*C. fpf* strain P990 plasmid pCff3	35.3%	22,293	circular
CP066342	*C.* sp. YC1 plasmid pCspYC1	67.0%	77,217	circular
CP071884	*C. fpf* strain BRIP:70614 plasmid pCff119	66.0%	119,821	linear
CP074440	*C. fpf* strain BRIP:70606 plasmid pCff119	66.0%	119,808	linear
CP081962	*C.* sp. TC1 plasmid pTCL	65.6%	163,762	circular
CP081963	*C.* sp. TC1 plasmid pTCS	67.8%	41,985	circular

**Table 4:** Primers and PCR product sequence for the genus-specific detection of *Curtobacterium*.

Name	Sequence	Tm	Product Size
Curto-F2	GAAATGGTGTTATGGCCGGAT	61.5 °C	275 bp
Curto-D-R	ACGGGTTAACCTCGCCACA	61.5 °C	
Product Sequence			
GAAATGGTGTTATGGCCGGATGTGTATCCCAAGTAGCACGGGGCCCGAGAAATCCCGTGTGAATCTGTCAGGACCACCTGATAAGCCTAAATACTCCCAGATGACCGATAGCGGACAAGTACCGTGAGGGAAAGGTGAAAAGTACCCCGGGAGGGGAGTGAAATAGTACCTGAAACCGTTTGCTTACAAACCGTCGGAGCCTCCTTGTAGGGGTGACGGCGTGCCTTTTGAAGAATGAGCCTGCGAGTTAGTGATATGTGGCGAGGTTAACCCGT			

**Table 5:** Detection sensitivity of the test plasmid and genomic DNA.

N°	Plasmid			Genomic DNA		
	Concentration	Mean Cq	SD	Concentration	Mean Cq	SD
1	2.18 × 10^9^	6.76	0.45	2.59 × 10^6^	18.61	0.03
2	2.18 × 10^8^	8.5	0.27	2.59 × 10^5^	24.01	0.09
3	2.18 × 10^7^	11.82	0.54	2.59 × 10^4^	27.95	0.23
4	2.18 × 10^6^	16.21	1.9	2.59 × 10^3^	29.93	0.08
5	2.18 × 10^5^	20.1	0.33	2.59 × 10^2^	32.66	0.09
6	2.18 × 10^4^	23.95	0.01	25.9	-	-
7	2.18 × 10^3^	26.37	0.01	2.59	-	-
8	2.18 × 10^2^	28.33	0.03	-	-	-
9	21.8	-	-	-	-	-

## Supplementary Materials

The following are available online at https://www.mdpi.com/article/10.3390/cimb44020060/s1: Figure S1: ANI matrix using 190 *Curtobacterium* genomes obtained with orthoANIu and clustered using BioNJ. Figure S2: Consensus tree obtained with MEGA using 16S rRNA genes of representatives of *Curtobacterium* and *Gryllotalpicola ginnsengisoli* DSM 22003 used as an outgroup. Bootstrap support values are shown near their branches as a percentage of 1000 replicates. The branches with bootstrap support of less than 50% were collapsed. Figure S3: Best-scoring tree obtained with RAxML using 16S rRNA genes of representatives of *Curtobacterium* and *Gryllotalpicola ginnsengisoli* DSM 22003 used as an outgroup. Bootstrap support values are shown near their branches as a percentage of 1000 replicates. The scale bar shows 0.005 estimated substitutions per site. Figure S4: Consensus tree obtained with MEGA using 23S rRNA genes of representatives of *Curtobacterium* and *Gryllotalpicola ginnsengisoli* DSM 22003 used as an outgroup. Bootstrap support values are shown near their branches as a percentage of 1000 replicates. The branches with bootstrap support of less than 50% were collapsed. Figure S5: Best-scoring tree obtained with RAxML using 23S rRNA genes of representatives of *Curtobacterium* and *Gryllotalpicola ginnsengisoli* DSM 22003 used as an outgroup. Bootstrap support values are shown near their branches as a percentage of 1000 replicates. The scale bar shows 0.01 estimated substitutions per site. Figure S6: Consensus tree obtained with MEGA using concatenated 16S and 23S rRNA genes of representatives of *Curtobacterium* and *Gryllotalpicola ginnsengisoli* DSM 22003 used as an outgroup. Bootstrap support values are shown near their branches as a percentage of 1000 replicates. The branches with bootstrap support of less than 50% were collapsed. Figure S7: Best-scoring tree obtained with RAxML using concatenated 16S and 23S rRNA genes of representatives of *Curtobacterium* and *Gryllotalpicola ginnsengisoli* DSM 22003 used as an outgroup. Bootstrap support values are shown near their branches as a percentage of 1000 replicates. The scale bar shows 0.01 estimated substitutions per site. Figure S8: Consensus tree obtained with MEGA using *gyrB* nucleotide sequences of representatives of *Curtobacterium* and *Gryllotalpicola ginnsengisoli* DSM 22003 used as an outgroup. Bootstrap support values are shown near their branches as a percentage of 1000 replicates. The branches with bootstrap support lower than 50% were collapsed. Figure S9: Best-scoring tree obtained with RAxML using *gyrB* nucleotide sequences of representatives of *Curtobacterium* and *Gryllotalpicola ginnsengisoli* DSM 22003 used as an outgroup. Bootstrap support values are shown near their branches as a percentage of 1000 replicates. The scale bar shows 0.05 estimated substitutions per site. Figure S10: Consensus tree obtained with MEGA using *parE* nucleotide sequences of representatives of *Curtobacterium* and *Gryllotalpicola ginnsengisoli* DSM 22003 used as an outgroup. Bootstrap support values are shown near their branches as a percentage of 1000 replicates. The branches with bootstrap support of less than 50% were collapsed. Figure S11: Best-scoring tree obtained with RAxML using *parE* nucleotide sequences of representatives of *Curtobacterium* and *Gryllotalpicola ginnsengisoli* DSM 22003 used as an outgroup. Bootstrap support values are shown near their branches as a percentage of 1000 replicates. The scale bar shows 0.1 estimated substitutions per site. Figure S12: Consensus tree obtained with MEGA using *rpoA* nucleotide sequences of representatives of *Curtobacterium* and *Gryllotalpicola ginnsengisoli* DSM 22003 used as an outgroup. Bootstrap support values are shown near their branches as a percentage of 1000 replicates. The branches with bootstrap support of less than 50% were collapsed. Figure S13: Best-scoring tree obtained with RAxML using *rpoA* nucleotide sequences of representatives of *Curtobacterium* and *Gryllotalpicola ginnsengisoli* DSM 22003 used as an outgroup. Bootstrap support values are shown near their branches as a percentage of 1000 replicates. The scale bar shows 0.05 estimated substitutions per site. Figure S14: Consensus tree obtained with MEGA using *rpoB* nucleotide sequences of representatives of *Curtobacterium* and *Gryllotalpicola ginnsengisoli* DSM 22003 used as an outgroup. Bootstrap support values are shown near their branches as a percentage of 1000 replicates. The branches with bootstrap support of less than 50% were collapsed. Figure S15: Best-scoring tree obtained with RAxML using *rpoB* nucleotide sequences of representatives of *Curtobacterium* and *Gryllotalpicola ginnsengisoli* DSM 22003 used as an outgroup. Bootstrap support values are shown near their branches as a percentage of 1000 replicates. The scale bar shows 0.05 estimated substitutions per site. Figure S16: Consensus tree obtained with MEGA using *gyrB*, *parE*, *rpoB*, and *rpoB* concatenated nucleotide sequences of representatives of *Curtobacterium* and *Gryllotalpicola ginnsengisoli* DSM 22003 used as an outgroup. Bootstrap support values are shown near their branches as a percentage of 1000 replicates. The branches with bootstrap support lower than 50% were collapsed. Figure S17: Best-scoring tree obtained with RAxML using *gyrB*, *parE*, *rpoB*, and *rpoB* concatenated nucleotide sequences of representatives of *Curtobacterium* and *Gryllotalpicola ginnsengisoli* DSM 22003 used as an outgroup. Bootstrap support values are shown near their branches as a percentage of 1000 replicates. The scale bar shows 0.1 estimated substitutions per site. Figure S18: (**A**) Predicted structure of putative DNA polymerase from *C. fpf* P990 plasmid pCff1 obtained with Alphafold and refined with ReFOLD coloured based on a rainbow gradient scheme, where the N-terminus of the polypeptide chain is coloured blue, and the C-terminus is coloured red. (**B**) ModFOLD 3D view of per-residue accuracy of the model of putative DNA polymerase from *C. fpf* P990 plasmid pCff1. The model is coloured based on a rainbow gradient scheme, where the residues with the lowest predicted residue errors are coloured blue, and the residues with the highest predicted residue errors are coloured red. (**C**) ModFOLD residue error plot. Table S1: List of bacterial strains used for the PCR development. Table S2: List of *Curtobacterium* genomes deposited in the NCBI GenBank database, as of October 2021.
