# Supplementary figures and images for: Curtobacterium spp. and Curtobacterium flaccumfaciens: Phylogeny, Genomics-Based Taxonomy, Pathogenicity, and Diagnostics
Source: Curr Issues Mol Biol. 2022 Feb 11;44(2):889–927. doi: 10.3390/cimb44020060 (PMC8929003; doi:10.3390/cimb44020060)

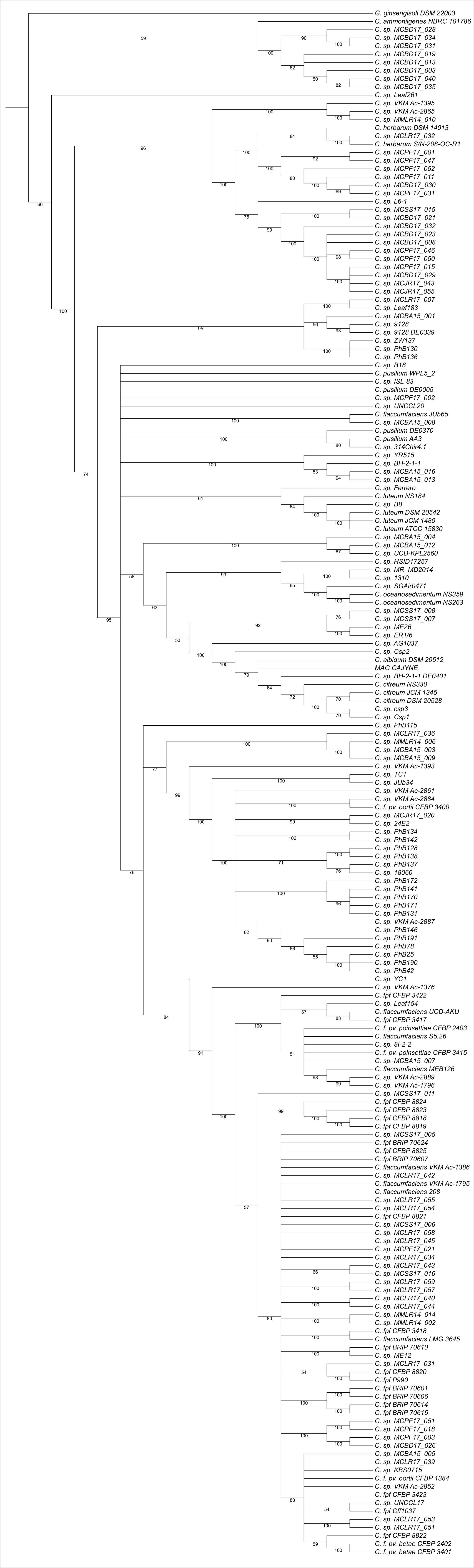

Supplement: Supplementary file 1 [file cimb-44-00060-s001.zip › Figure_S10-mega-topIV.jpg]

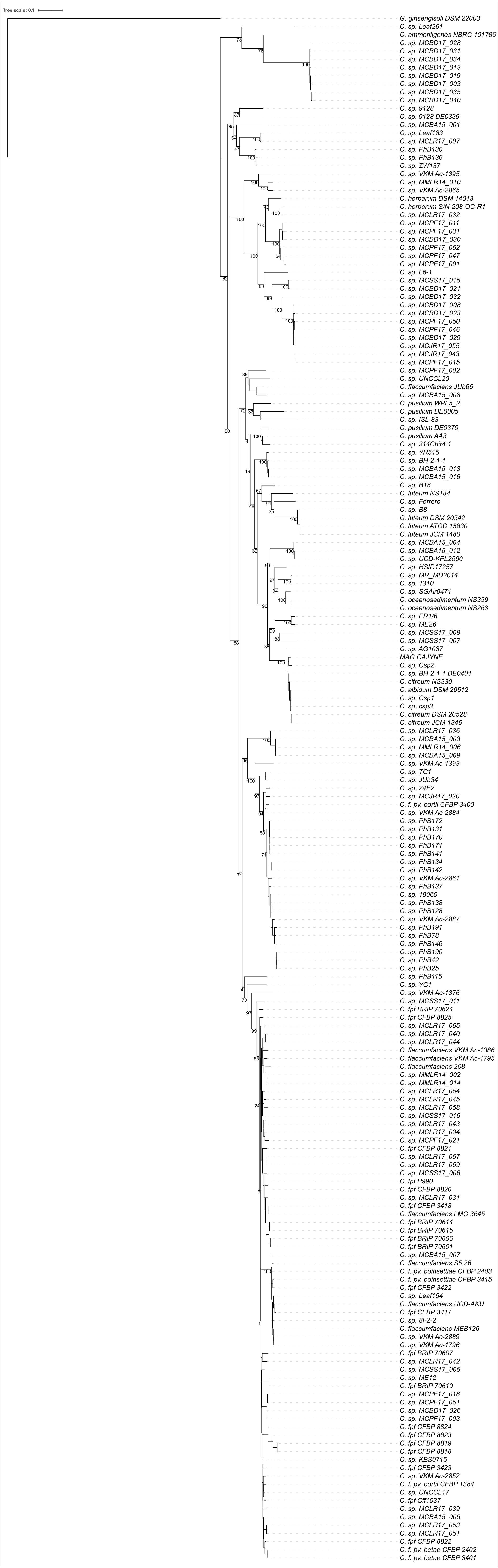

Supplement: Supplementary file 1 [file cimb-44-00060-s001.zip › Figure_S11-raxml-topIV.jpg]

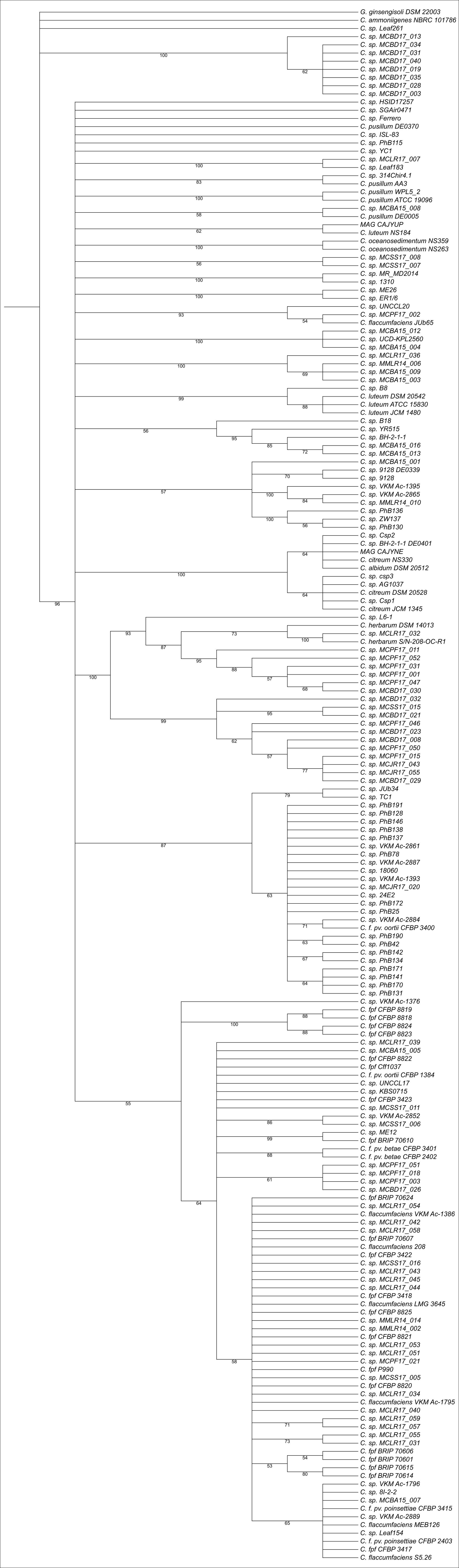

Supplement: Supplementary file 1 [file cimb-44-00060-s001.zip › Figure_S12-mega-rpoA.jpg]

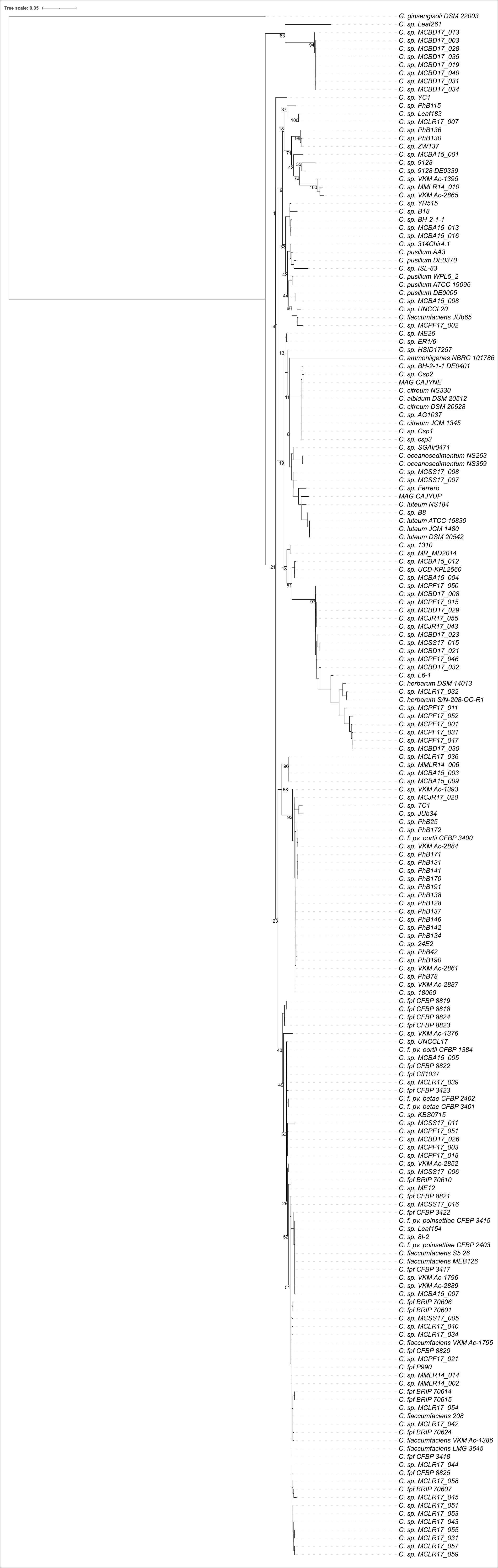

Supplement: Supplementary file 1 [file cimb-44-00060-s001.zip › Figure_S13-raxml-rpoA.jpg]

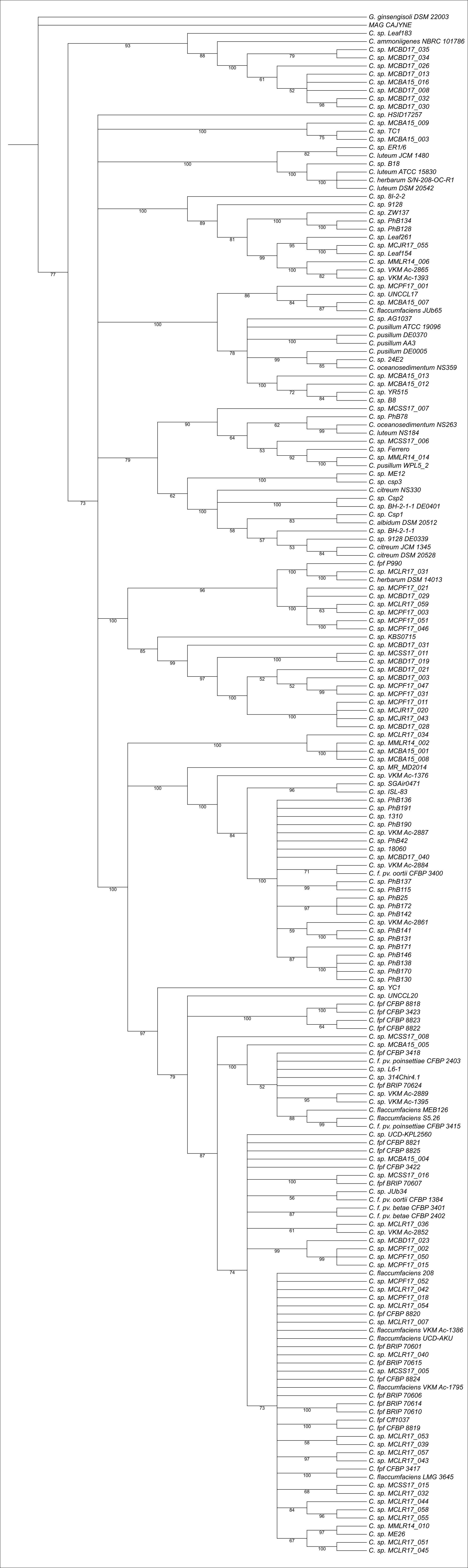

Supplement: Supplementary file 1 [file cimb-44-00060-s001.zip › Figure_S14-mega-rpoB.jpg]

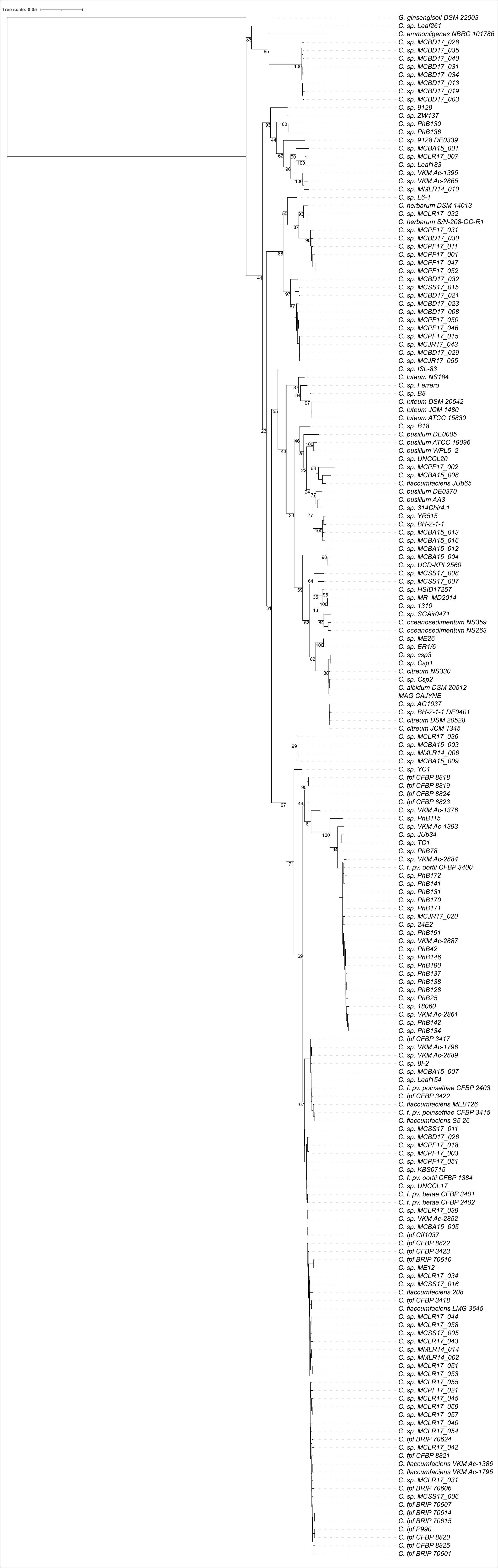

Supplement: Supplementary file 1 [file cimb-44-00060-s001.zip › Figure_S15-raxml-rpoB.jpg]

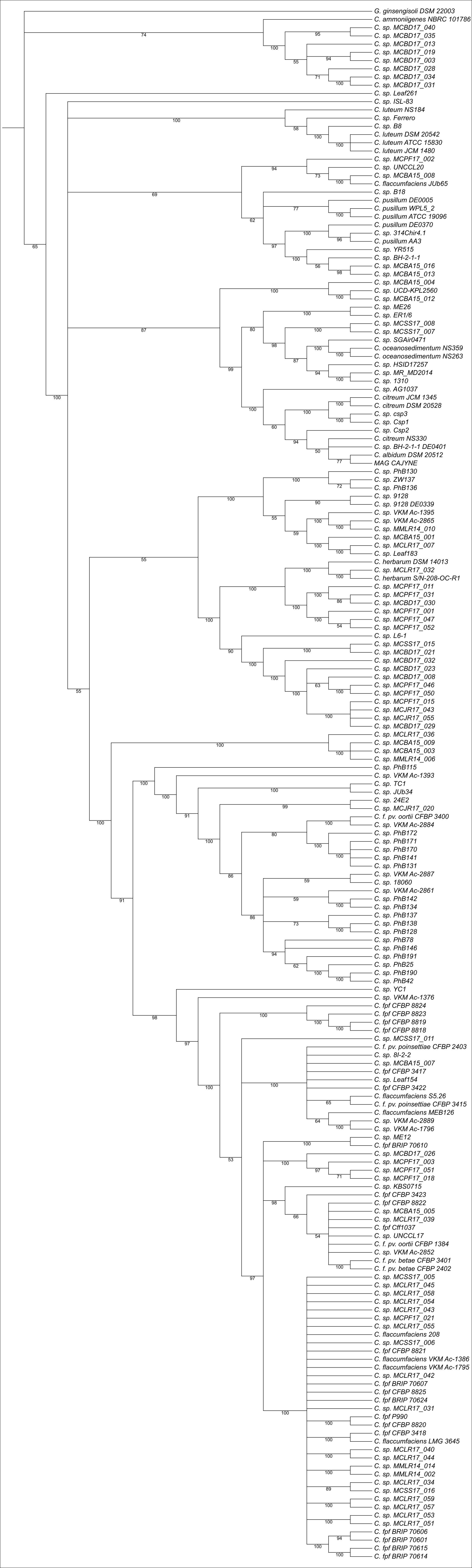

Supplement: Supplementary file 1 [file cimb-44-00060-s001.zip › Figure_S16-mega-mlst-4.jpg]

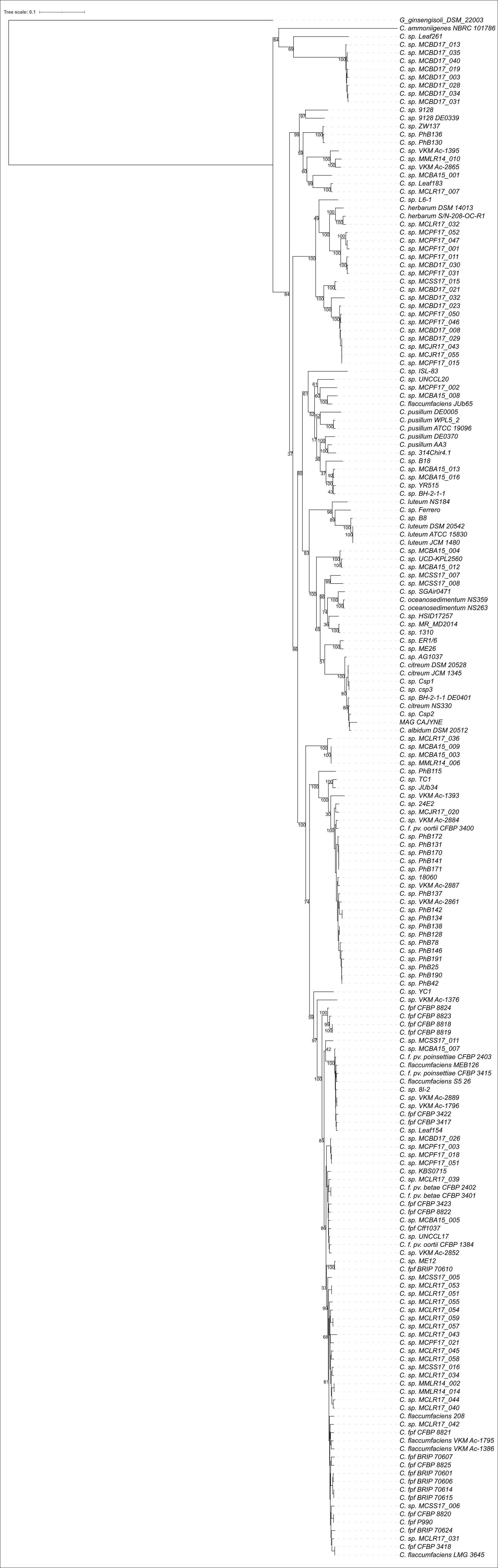

Supplement: Supplementary file 1 [file cimb-44-00060-s001.zip › Figure_S17-raxml-mlst-4.jpg]

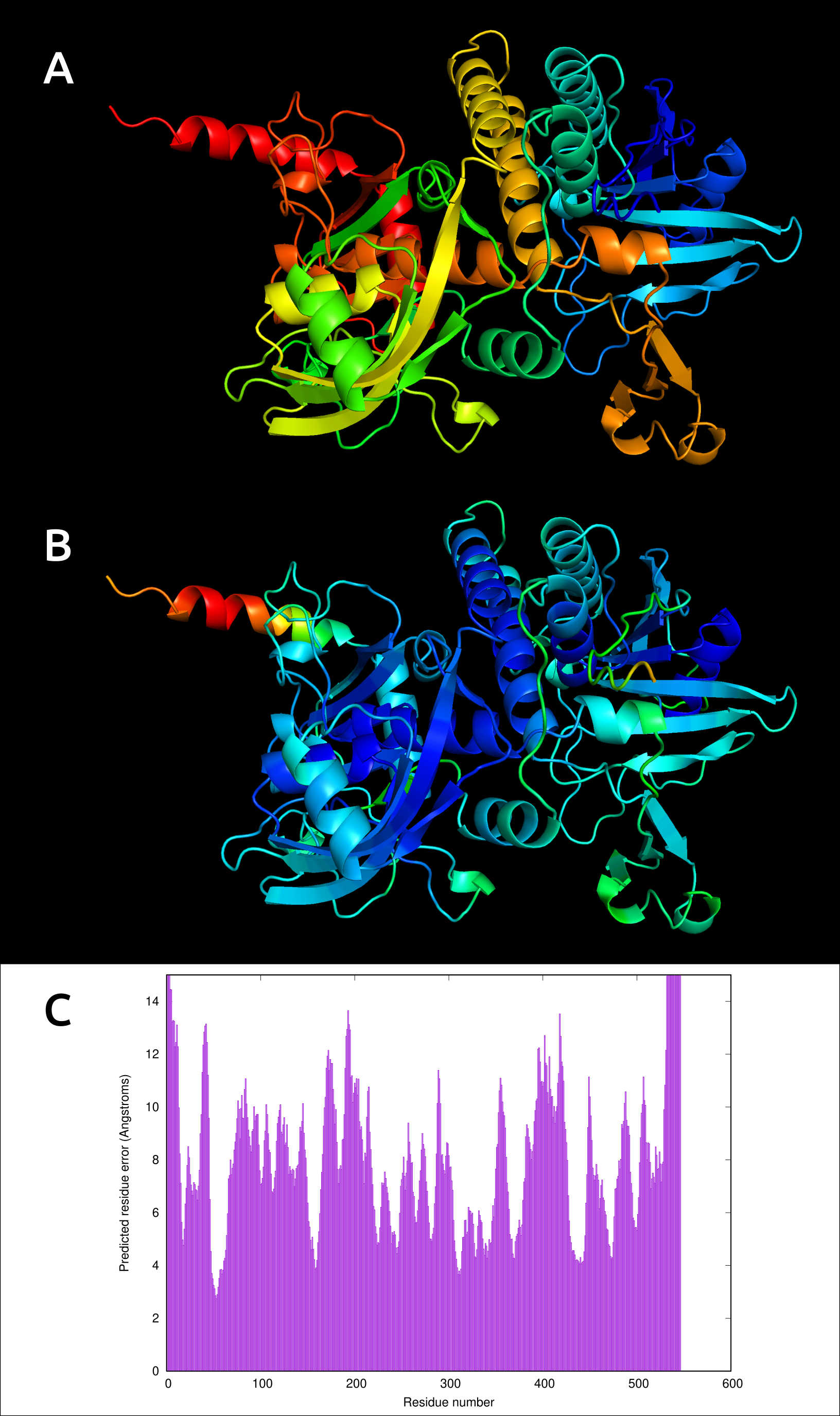

Supplement: Supplementary file 1 [file cimb-44-00060-s001.zip › Figure_S18.jpg]

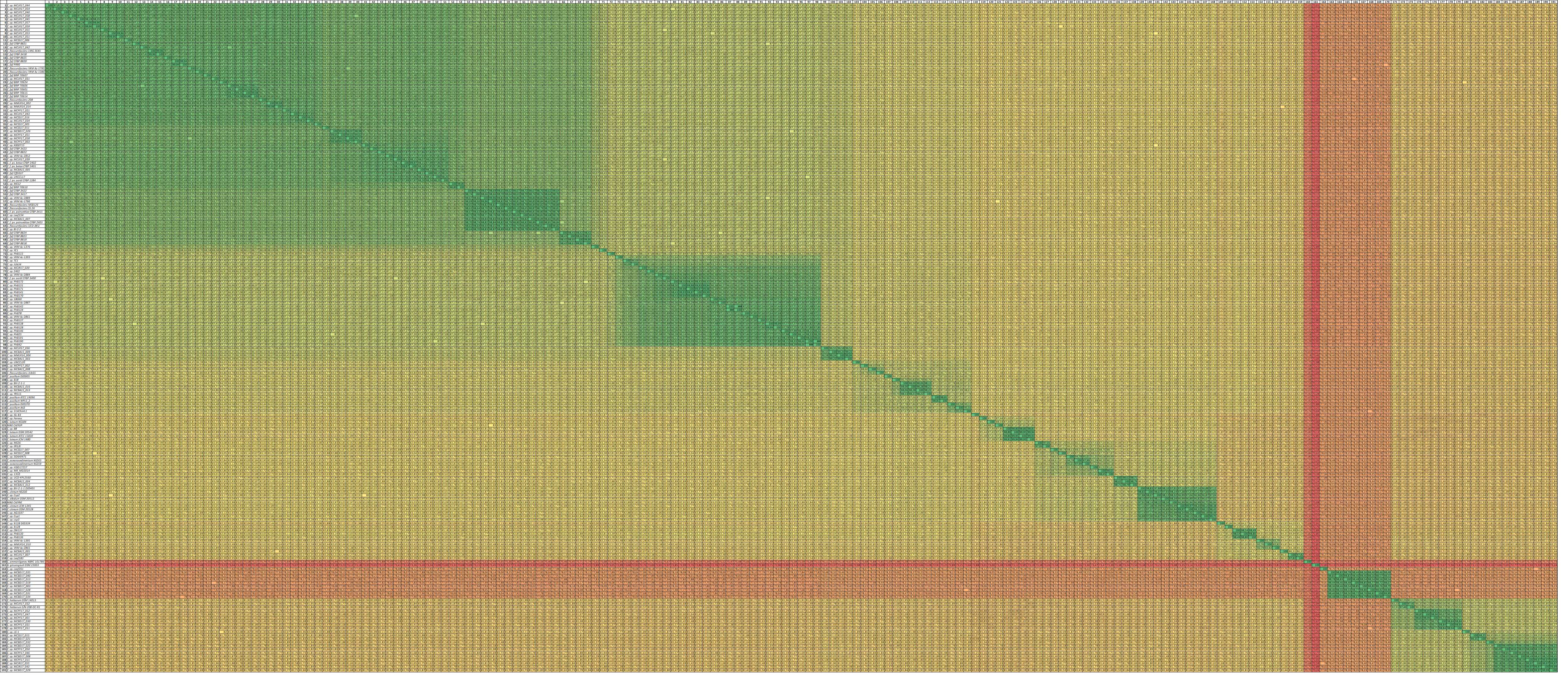

Supplement: Supplementary file 1 [file cimb-44-00060-s001.zip › Figure_S1_ANI_BioNJ_matrix.jpg]

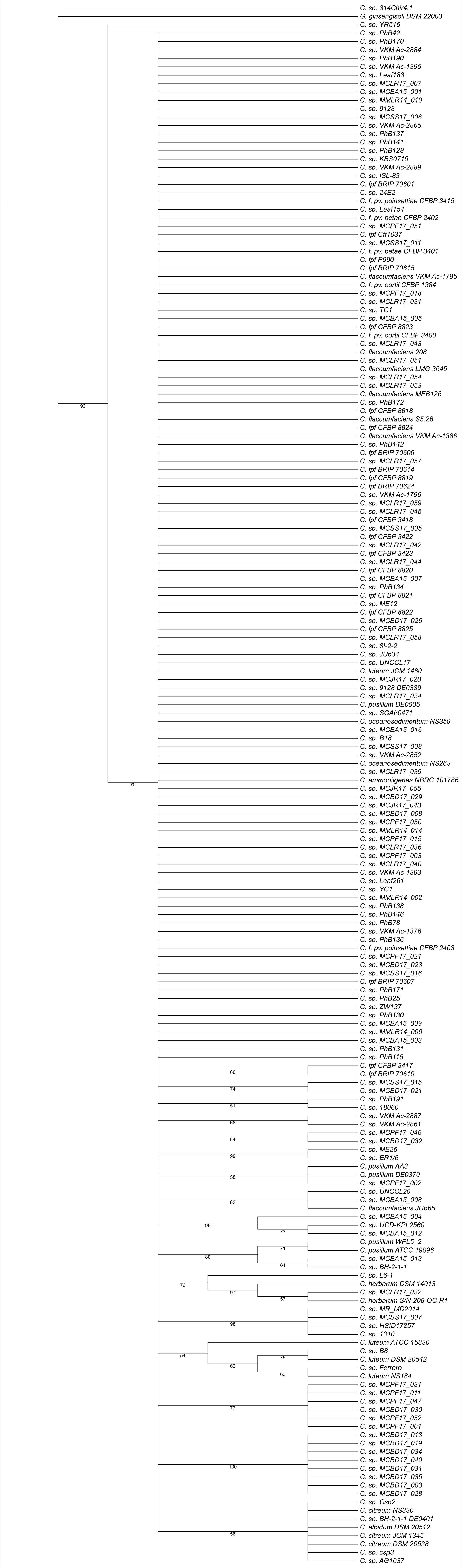

Supplement: Supplementary file 1 [file cimb-44-00060-s001.zip › Figure_S2-mega-16S.jpg]

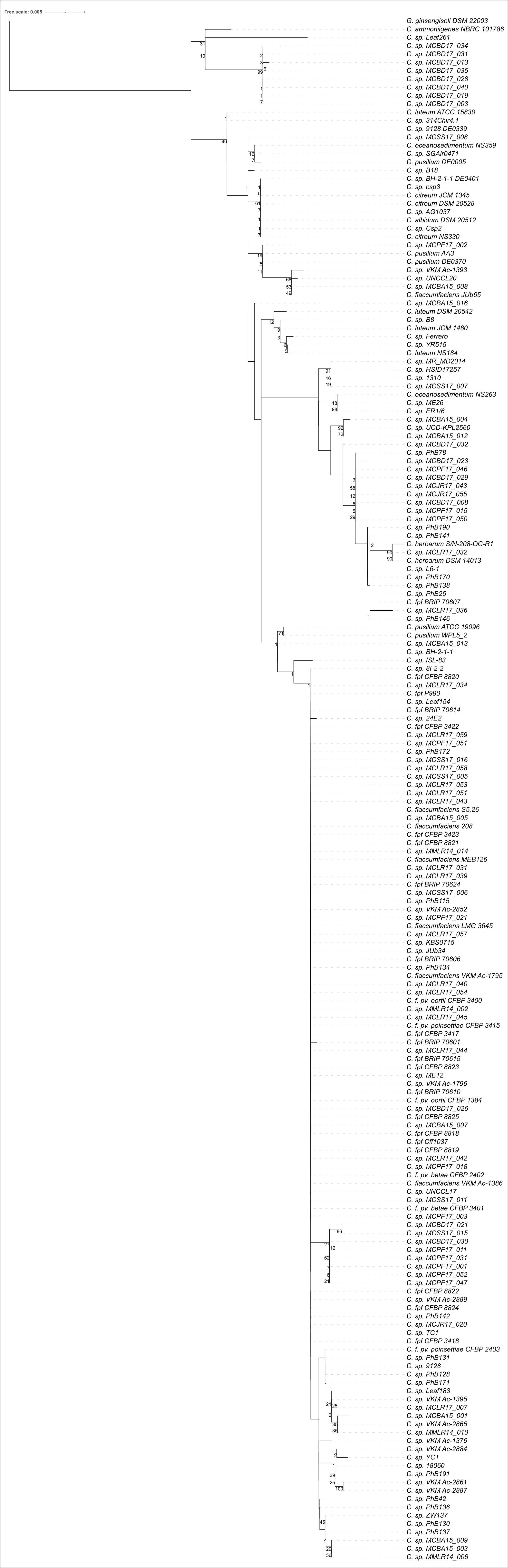

Supplement: Supplementary file 1 [file cimb-44-00060-s001.zip › Figure_S3-raxml-16S.jpg]

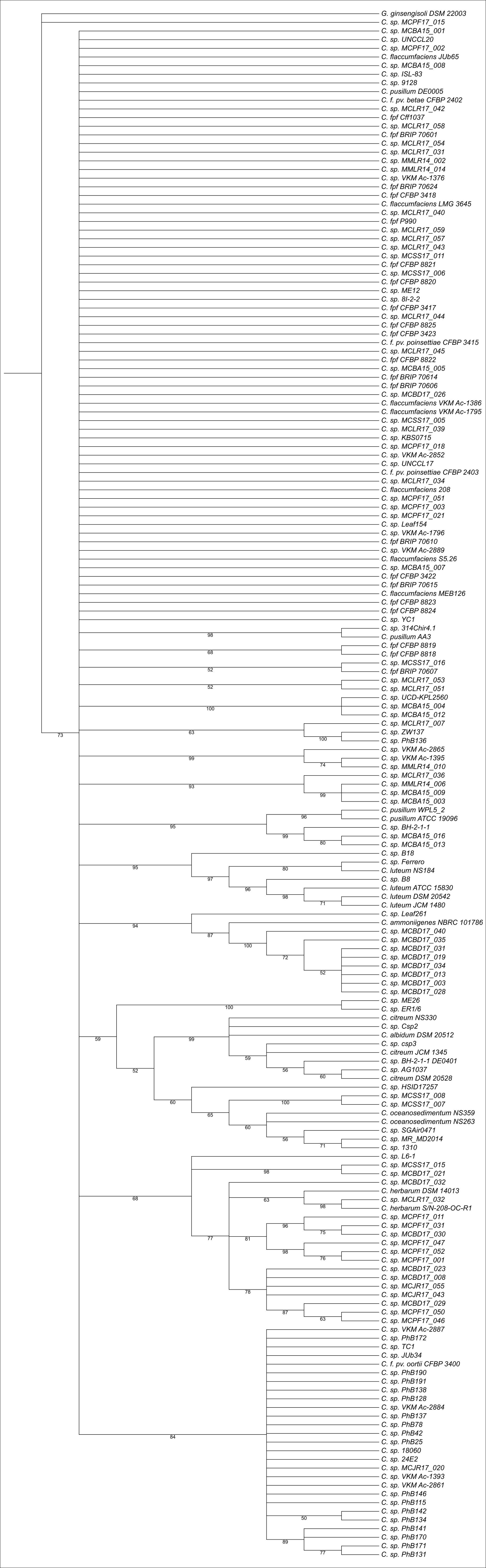

Supplement: Supplementary file 1 [file cimb-44-00060-s001.zip › Figure_S4-mega-23S.jpg]

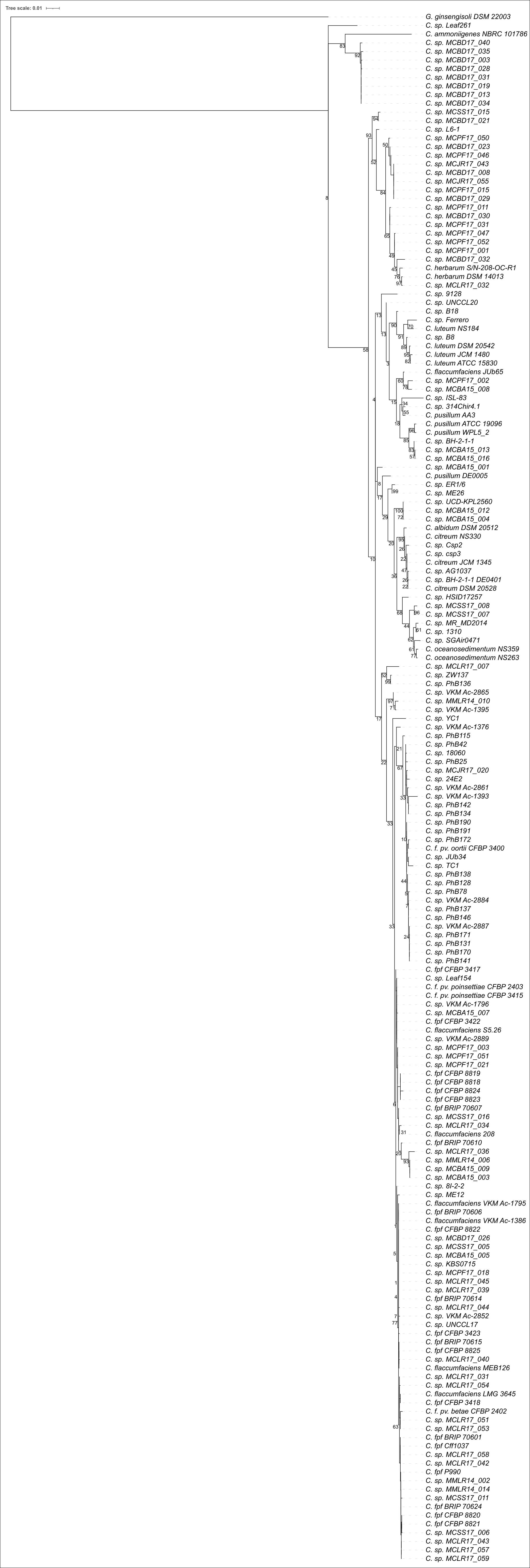

Supplement: Supplementary file 1 [file cimb-44-00060-s001.zip › Figure_S5-raxml-23S.jpg]

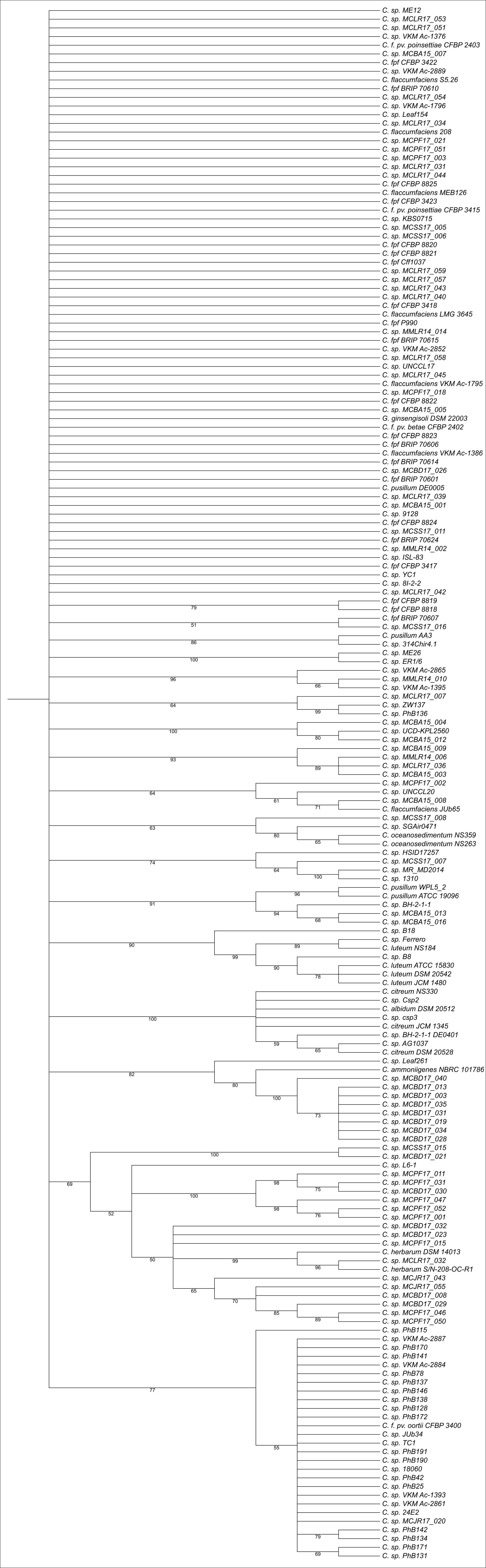

Supplement: Supplementary file 1 [file cimb-44-00060-s001.zip › Figure_S6-mega-16S-23S.jpg]

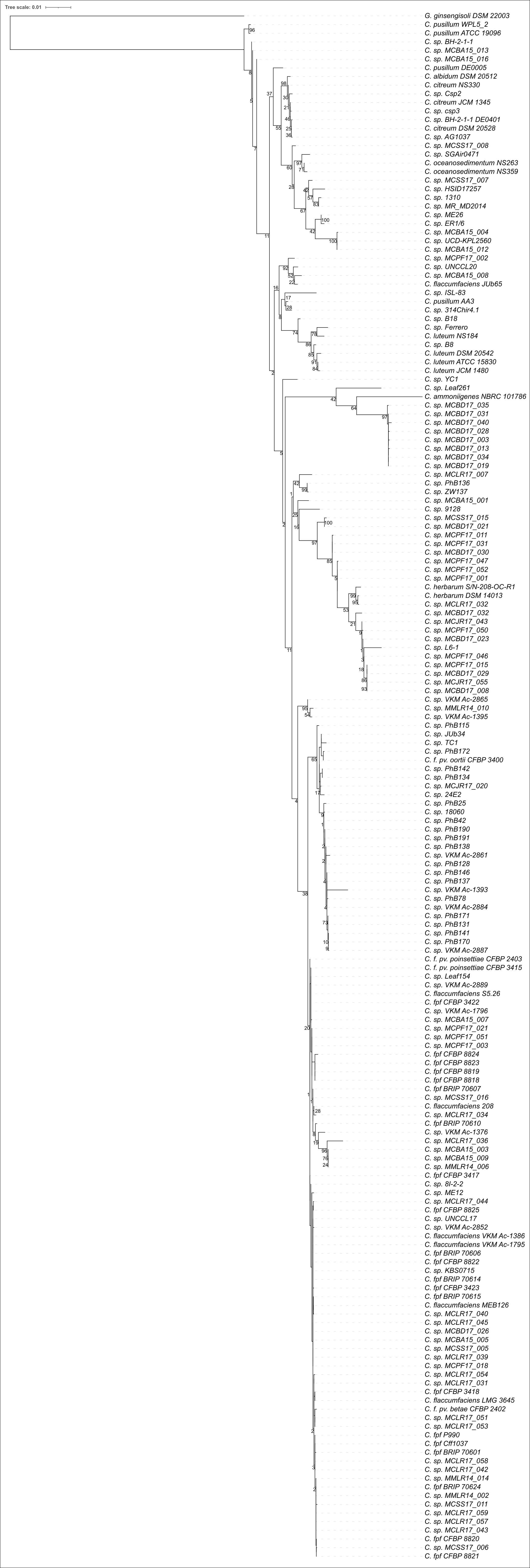

Supplement: Supplementary file 1 [file cimb-44-00060-s001.zip › Figure_S7-raxml-16S-23S.jpg]

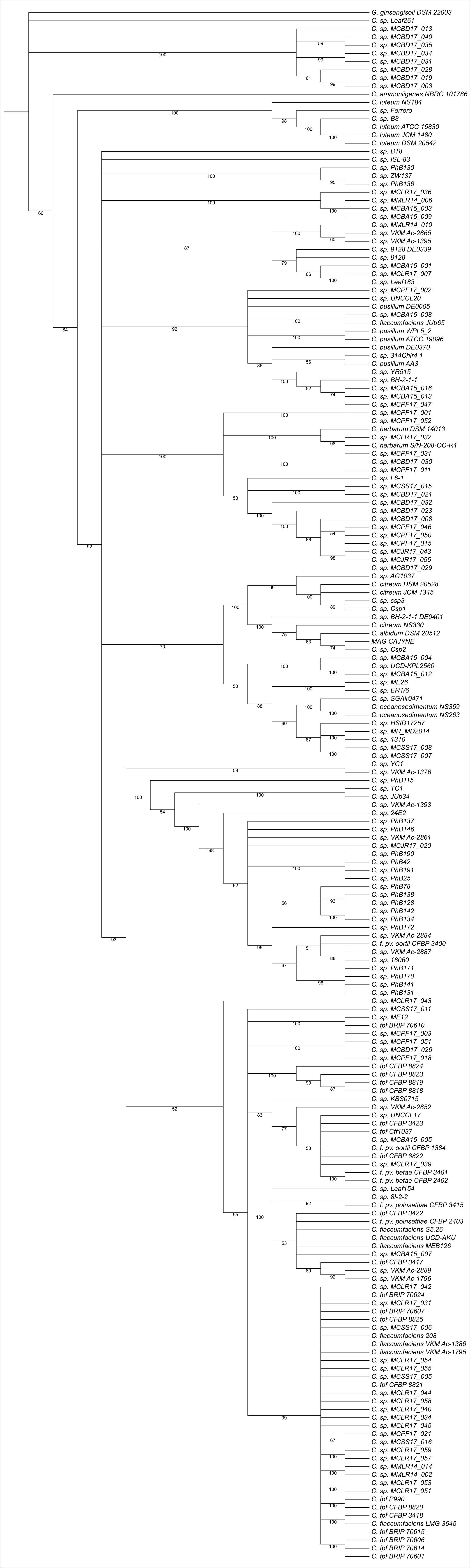

Supplement: Supplementary file 1 [file cimb-44-00060-s001.zip › Figure_S8-mega-gyrB.jpg]

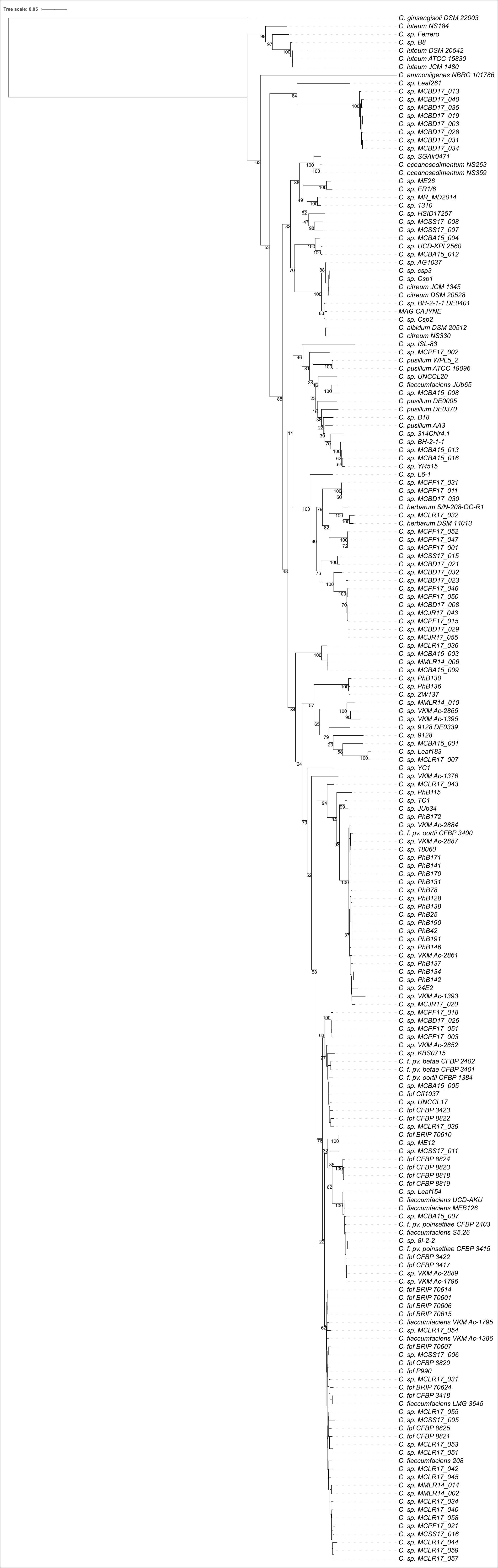

Supplement: Supplementary file 1 [file cimb-44-00060-s001.zip › Figure_S9-raxml-gyrB.jpg]
